# Non-Invasive microRNA Profiling in Saliva can Serve as a Biomarker of Alcohol Exposure and Its Effects in Humans

**DOI:** 10.3389/fgene.2021.804222

**Published:** 2022-01-20

**Authors:** Edward A. Mead, Nadia Boulghassoul-Pietrzykowska, Yongping Wang, Onaiza Anees, Noah S. Kinstlinger, Maximillian Lee, Shireen Hamza, Yaping Feng, Andrzej Z. Pietrzykowski

**Affiliations:** ^1^ Laboratory of Adaptation, Reward and Addiction, Department of Animal Sciences, Rutgers University, New Brunswick, NJ, United States; ^2^ Department of Genetics and Genomic Sciences, Icahn School of Medicine at Mount Sinai, New York, NY, United States; ^3^ Mayo Clinic Health System, NWWI, Barron, WI, United States; ^4^ Department of Medicine, Capital Health, Trenton, NJ, United States; ^5^ Weight and Life MD, Hamilton, NJ, United States; ^6^ Holmdel Township School, Holmdel, NJ, United States; ^7^ Virginia Commonwealth University Health, CMH Behavioral Health, South Hill, VA, United States; ^8^ Albert Einstein College of Medicine, Bronx, NY, United States; ^9^ George Washington University, School of Medicine and Health Sciences, Washington DC, MA, United States; ^10^ Department of the History of Science, Harvard University, Cambridge, MA, United States; ^11^ Waksman Genomics Core Facility, Rutgers University, Piscataway, NJ, United States; ^12^ Bioinformatics Department, Admera Health, South Plainfield, NJ, United States

**Keywords:** abuse, alcohol, array, microRNA, profiling, saliva, biomarker

## Abstract

Alcohol Use Disorder (AUD) is one of the most prevalent mental disorders worldwide. Considering the widespread occurrence of AUD, a reliable, cheap, non-invasive biomarker of alcohol consumption is desired by healthcare providers, clinicians, researchers, public health and criminal justice officials. microRNAs could serve as such biomarkers. They are easily detectable in saliva, which can be sampled from individuals in a non-invasive manner. Moreover, microRNAs expression is dynamically regulated by environmental factors, including alcohol. Since excessive alcohol consumption is a hallmark of alcohol abuse, we have profiled microRNA expression in the saliva of chronic, heavy alcohol abusers using microRNA microarrays. We observed significant changes in salivary microRNA expression caused by excessive alcohol consumption. These changes fell into three categories: downregulated microRNAs, upregulated microRNAs, and microRNAs upregulated *de novo*. Analysis of these combinatorial changes in microRNA expression suggests dysregulation of specific biological pathways leading to impairment of the immune system and development of several types of epithelial cancer. Moreover, some of the altered microRNAs are also modulators of inflammation, suggesting their contribution to pro-inflammatory mechanisms of alcohol actions. Establishment of the cellular source of microRNAs in saliva corroborated these results. We determined that most of the microRNAs in saliva come from two types of cells: leukocytes involved in immune responses and inflammation, and buccal cells, involved in development of epithelial, oral cancers. In summary, we propose that microRNA profiling in saliva can be a useful, non-invasive biomarker allowing the monitoring of alcohol abuse, as well as alcohol-related inflammation and early detection of cancer.

## Introduction

Alcohol is the oldest addictive substance. Currently, Alcohol Use Disorder (AUD) is among the most prevalent mental disorders in the world (Grant et al., 2007). In the United States, excessive alcohol consumption is the third leading cause of preventable death (NIH-NIAAA, 2021a). Over ninety-five thousand people die every year from the consequences of alcohol consumption, which includes alcohol-related illnesses and accidents (Centers for Disease Control and Prevention, 2020). Moreover, with 14% of alcohol users suffering from alcohol dependence and over $249 billion of estimated alcohol-related costs in 2010 alone, AUD puts a substantial economic burden on the society (National Institutes of Health, 2021a). Excessive alcohol consumption affects both men and women of various ages and diverse ethnic groups (National Institutes of Health, 2021a). The current fifth edition of the Diagnostic and Statistical Manual of Mental Disorders (DSM-5) classifies AUD based on its severity into several categories under the label of alcohol use disorder: mild, moderate and severe (Gruenberg, 2013; National Institutes of Health, 2021b). A key diagnostic and pathogenic criterion is an exposure to alcohol. While the labeling has changed in development of DSM-5, essentially the same core criteria exists: patients suffering from AUD drink often and in excess, despite negative consequences caused or exacerbated by drinking (National Institutes of Health, 2021b). Nationwide statistics indicate that, as of 2019, a staggering 14.5 million Americans 12 years old and up were consuming alcohol in a pathological manner (National Institutes of Health, 2021a).

Considering the widespread occurrence of AUD, a reliable biomarker of pathological alcohol consumption would be useful to healthcare providers, clinicians, researchers, as well as public safety workers or those in the criminal justice system (Litten et al., 2010; Nanau and Neuman, 2015). This biomarker should also be inexpensive and easy to use at varying socioeconomic levels. Ability to perform frequent sampling of the same individuals would permit monitoring of progress of the disease with its many diverse features like escalation of drinking, development of tolerance, abstinence or relapse, from mild to severe subtypes of AUD (National Institutes of Health, 2021b). Several compounds have been considered, or are currently used, as alcohol biomarkers (see reviews by Litten et al., 2010; Nanau and Neuman, 2015). Traditional and new biomarkers are mainly alcohol metabolites, or direct or indirect indicators of abnormal enzymatic activity. Carbohydrate-deficient transferrin (CDT) and ethyl glucuronide (EtG) are two particularly promising biomarkers due to their high sensitivity and specificity. However, their measurements still require costly methodology: high performance liquid chromatography (HPLC) to determine CDT, and liquid chromatography mass spectroscopy (LC-MS) to measure EtG (Litten et al., 2010; Nanau and Neuman, 2015).

microRNAs are intracellular molecules, belonging to a class of small, non-coding RNAs, (Stickel and Dubuquoy, 2016). microRNA binds to its mRNA target causing either mRNA degradation or impairment of mRNA translation (Lim et al., 2005; Peterson et al., 2014). Since a single microRNA can bind to several mRNA transcripts, one microRNA can simultaneously regulate expression of hundreds of mRNA targets. Moreover, efficiency of microRNA binding can vary from target to target, allowing microRNA to regulate gene expression with great finesse. microRNAs are estimated to modify expression of over 60% of the human transcriptome (Shu et al., 2017) making microRNAs very powerful post-transcriptional regulators of overall gene expression.

Because many microRNAs are differentially expressed in pathological cells compared to normal cells, attempts are being made to use microRNAs as biomarkers in several diseases, *e.g*., in neurological disorders (Shafi et al., 2010; Wang et al., 2021) cancer (Vu et al., 2021), infections (Wang et al., 2016), aging (Kumar et al., 2017) and forensics (Silva et al., 2015). Interestingly, microRNAs emerge as attractive biomarkers of cancer as microRNA profiling seems to more accurately cluster different types of cancer rather than mRNA (Lu et al., 2005). (Gilad et al., 2008; Yang et al., 2018). It is tempting to consider microRNAs as an attractive alternative to traditional markers of alcohol consumption in AUD (Rosato et al., 2019; Lim et al., 2021).

Alcohol has a global effect on the body and its regulation of microRNAs has been shown in different organs and cell types like the liver (Bala and Szabo, 2012; Dippold et al., 2013), gastrointestinal tissue (Tang et al., 2008; Natarajan et al., 2015), the brain (Lewohl et al., 2011; Natarajan et al., 2015) and others (Natarajan et al., 2015).

De-regulation of leukocytes by alcohol can contribute to the immunosuppressive and inflammatory effects of alcohol. The association between the development of cancer and inflammatory processes has been well appreciated (Coussens and Werb, 2002; Greten and Grivennikov, 2019). Moreover, carcinogenic effects of alcohol, particularly chronic alcohol exposure, are also thought to take place, at least in part, *via* modulation of inflammatory responses (Seitz and Stickel, 2007; Ureña-Peralta et al., 2018). Specifically, cancers originating from the epithelial cells seem to be affected by alcohol (Vagst et al., 2003; Gupta et al., 2010; Michaud et al., 2010; Fedirko et al., 2011; Johnson et al., 2011; Pelucchi et al., 2011; Meadows and Zhang, 2015).

Since research has shown that microRNAs are present in saliva (Park et al., 2009; Wong, 2015; Rosato et al., 2019; Romani et al., 2021) and it is a minimally invasive, non-biohazardous biofluid to collect, saliva can be an attractive source of biomarkers for AUD, as well as related disorders including inflammation and cancer.

The aim of this study was to establish whether alcohol affects microRNA expression in saliva, and to demonstrate feasibility of using microRNA profiling in saliva as a biomarker of chronic, excessive alcohol consumption. We also aimed to determine potential functional consequences of microRNA expression altered by alcohol, providing further insight into pathogenesis of AUD.

## Materials and Methods

### Subject Selection

All patients selected to this study were admitted to Capital Health Hospital, Fuld Campus, Trenton, NJ due to underlying medical condition requiring hospitalization (e.g., pneumonia, acute kidney injury, chronic kidney disease secondary to hypertension, secondary diabetes mellitus, chest pain, ischemic heart disease, congestive heart failure or cardiac arrhythmia). All patients were adults over 21 years old, enrolled without restrictions based upon race, ethnicity or gender, living locally (within approximately 50 miles from the hospital). Since the Capital Health Hospital, Fuld Campus covers a large Polish community, in addition to the English consent form, a certified Polish consent form was also available, as well as a Polish-speaking provider. Thus, inclusion criteria included individuals able to fully communicate in English and/or Polish.

Individuals were selected based on their alcohol drinking pattern, which was determined based on a number of drinking bouts per week, a number of drinks per bout and an overall drinking period. Two groups of individuals were considered in this study: 1/controls, which drank alcohol sporadically and in low amounts with an average alcohol consumption: 16.4 gm EtOH/24 h (non-abusers), and 2/heavy drinkers with at least a ten year history of daily alcohol consumption, with current alcohol intake of 230 gm EtOH/24 h on average (abusers). A standard drink was defined as 14 g of ethanol, which is equivalent to either twelve ounces of beer, five ounces of wine or one ounce of distilled spirits (Ferreira and Willoughby, 2008). Controls consumed alcohol sporadically: not more often than once a week and not more than one drink. In order to identify suitable subjects, 1351 medical records were reviewed, from which 330 subjects were selected for an interview, which focused on determination of the alcohol drinking pattern based on DSM-IV. 202 patients were selected and presented with a thorough explanation of the study and the written consent form. 37 subjects elected to participate in the study.

Exclusion criteria included: minors (<21 years of age), patients with intellectual disability, or conditions not allowing for proper communication (*e.g*., aphasia), non-English/non-Polish speaking individuals, and patients with known drug abuse history, including nicotine addiction (smokers). Non-smokers were permitted to participate in the study. This study (including a bilingual consent form) was approved by two Ethical Review Board Committees (Capital Health Hospital System, and Rutgers State University of New Jersey**).**

### Sample Collection

Saliva was collected using the Oragene RNA collection kits (DNA Genotek Inc., Canada) from all patients enrolled in this study during their hospitalization no earlier than 48 h after hospitalization. Typically, saliva samples were collected on the day of discharge, when any active acute sickness for which patients were admitted was resolved, and consent forms were explained and accepted when patients were fully sober. All saliva samples were self-collected by patients under the supervision of a physician following the manufacturer’s protocol. Patients did not eat or drink for an hour prior to saliva collection. Two (2) ml of saliva were collected directly into collection tubes without any stimulation of saliva secretion or scraping of cheeks. Tubes were capped immediately and mixed thoroughly with the self-releasing container cap stabilization solution, which also neutralizes SARS-CoV-2 virus responsible for COVID-19 (DNA Genotek, 2021).

### RNA Isolation

For each sample, total RNA was isolated using mirVana miRNA isolation kit following manufacturer guidelines (Life Technologies, NY) with minor modifications. RNA was stored immediately at −80°C to preserve its integrity until further analysis. Briefly, 500 μL aliquot of each saliva sample from the last step was incubated at 50°C overnight and the next day the aliquot was incubated at 90°C for 15 min miRNA homogenate additive, in volumes of 1/10, was then added in each aliquot. An equal volume of acid-phenol:chloroform was added to each aliquot and mixed well. The mixtures were spun for 10 min at 10,000 × g. Next, 1.25 volumes of 100% ethanol were added to the aqueous phase. The mixture was passed through a mirVana column in sequential 700-μL aliquots. Several washing steps were carried out. Finally, RNA was recovered in 50 μL of elution buffer. The eluted RNAs were treated with DNase I to remove DNA from RNA (Life Technologies, NY). The RNA concentration was quantified using a NanoDrop 1000 (NanoDrop Technologies, DE). RNA integrity and quality control were additionally measured for all samples on an Agilent 2100 Bioanalyzer (Agilent Technologies, CA).

### microRNA Expression Profiling Using TaqMan Low-Density Array

Tested subjects were not pooled, but rather an individual array was used for each subject. Thus, thirty-seven stem-loop RT-PCR based TaqMan Human microRNA Taqman low-density arrays were used (v3.0, Applied Biosystems, Foster City, CA). Each array consisted of two cards (Card A and B) with 384 TaqMan MicroRNA Assays per card. Arrays were designed based on a set of human-specific, mature microRNAs present in the Sanger miRBase v14 (http:/www.mirbase.org/). In addition, each card contains four control microRNAs including three carefully selected candidate endogenous controls for data normalization, inter- and intra-array control as well as a negative control (a Drosophila-specific microRNA).

After isolation of total RNA from saliva, all mature miRNAs were reverse transcribed into complementary DNA (cDNA) using looped-primer RT-PCR according to the manufacturer’s instructions. All reagents were obtained from Applied Biosystems. Three µl of total RNA were reverse transcribed using the TaqMan miRNA RT kit in combination with Megaplex RT primers in a total volume of 7.5 µL. The cDNA templates were pre-amplified using Megaplex Pre-Amp Primers and TaqMan Pre-Amp Master Mix. Twenty-seven µl of the 1:4 diluted preamplified product were amplified using sequence-specific primers and probes on the Taqman MicroRNA Array. PCR was performed using the 7900HT Fast Real-Time PCR (Applied Biosystems, CA) or Viia 7 Real-Time PCR thermocycler systems (Applied Biosystems, CA) following manufacturer’s recommended cycling conditions. Since each array results correspond to a separate subject, they were analyzed individually using the Applied Biosystems generic ExpressionSuite Software (v1.0.2) with standard settings containing FDR correction. Cycle threshold (C_t_) values were calculated using automatic baseline settings and a threshold of 0.2. GeNorm (Vandesompele et al., 2002) was used initially to determine control miRNAs. MammU6 was used as an endogenous control because of its low C_t_ values and very low variability amongst samples. C_t_ values >37 were considered to be below the detection level. miRNAs present in at least 50% of the control measurements were included in the analysis. Relative miRNA expression was calculated using the ΔΔCt method. The results were expressed as a fold change, unless stated otherwise. The Mann-Whitney *U* test was performed to detect differentially expressed miRNAs between groups (alcohol abusers vs non-abusing controls) using GraphPad (v6.0a). *p* < 0.05 was set as significant.

### Quantitative Real-Time PCR Analysis of Individual miRNAs

Real-Time quantitative PCR (qPCR) was performed by Taqman assay according to manufacturer’s instructions for Small RNA assays (Applied Biosystems, Foster City, CA). Ten individual microRNAs (see below) across the upregulated, expression *de novo*, downregulated, and unchanged microRNA categories were analyzed with U6 as a control from individual samples taken from each of six of individuals (*n* = 6) at random who had been screened by microarray card (3 controls, 3 alcohol abusers) and individually compared against the results for the same individuals from the arrays. miRNAs examined include hsa-miR-1, -10a-5p, -182-5p, -26a-5p, -27a-3p, -20a-5p, -29c-3p, -106a-5p, -9-5p, -618. Please note that microRNAs are named according to the nomenclature of miRBase 22.1 in which mature microRNAs coming from the complementary strands of a precursor are called miR-xx-5p and -3p, respectively, not miR-xx and miR-xx*.

Supplies used included Taqman Universal PCR master mix II (2x) no UNG, Taqman microRNA assays for each of the miRNAs under analysis, and 1:4 dilutions of the pre-amplification cDNA prepared for the microarray card assays. In short, a tube containing enough Universal Master Mix and nuclease-free water to provide 10 ul and 7.67 ul respectively of each for each well was prepared. This was gently mixed and aliquoted into separate tubes for each miRNA (or to U6) under analysis, to allow for 20x Taqman miRNA assay (primers + probe) to be added to each tube (enough for 1ul of final Taqman miRNA assay/well corresponding to that miRNA or U6). These final master mix (FMM) tubes were aliquoted into the appropriate wells, and for each well, 1.33 ul of a 1:4 dilution of cDNA corresponding to a given individual was added to each appropriate well, as directed by Applied Biosystems. To ensure the greatest comparative accuracy for a given miRNA, all of the 6 individual cDNA samples were examined together on the same plate for a given miRNA at the same time. This also served as a good control measure for assessing the arrays, which required a separate card for each individual examined: if the array results were correct, and preparation to preparation differences were non-significant, then the miRNA qRT-PCR assays which could allow for examining all samples for a given miRNA together on the same plate, would agree with the array results. For each plate, MammU6 was used for normalization across plates. Each sample was analyzed in 3 experimental replicates (3 wells loaded with FMM + cDNA) to allow for accuracy verification. Altogether, 66 individual qPCR reactions were performed in the technical triplicate, to create replicates for qualitative analysis. Technical triplicate means that each reaction was repeated 3 times and the average calculated. Individual differences among 3 repeats were smaller than 1 Ct. Positive, negative and NoRT controls were included routinely into each PCR reaction.

qRT-PCR was performed on a StepOne Plus ABI thermocycler (Applied Biosystems). Standard settings were used, except as noted. For assays, ΔΔCt assay was selected. Standard ramp was used, and the run length was standard (i.e., not a “fast” reaction). 6-FAM was the reporter, and the quencher was NFQ-MGB. The reaction volume was 20 ul, and a standard 40 cycle run was conducted, though no 50C step was used at the beginning as the samples lacked UNG. Thus, the run consisted of: 95°C 10 min, followed by 40 cycles of (95°C 15 s, 60°C 1 min). Data collection occurred on the 60°C step at each cycle.

### DIANA mirPath and KEGG Analysis

Kyoto Encyclopedia of Genes and Genomes (KEGG) is a free database (Kanehisa and Goto, 2000), which provides information about gene content of biological pathways, interrelationship of these genes, and involvement of pathways in pathogenesis of diseases. Data are supplied as tables and wiring diagrams of interactive networks. Analysis of KEGG pathways regulation by microRNA was performed using DIANA mirPath (v1.0 and v2.0), based on high-accuracy target prediction algorithms: DIANA-microT-CDS (Papadopoulos et al., 2009) and TargetScan (Garcia et al., 2011) with FDA correction (Benjamini and Hochberg, 1995). DIANA mirPath is a free web-based computational tool, developed by DNA Intelligent Analysis Laboratory (DIANA) that identifies biological pathways potentially altered by the expression of a single or multiple microRNAs (Papadopoulos et al., 2009; Vlachos et al., 2015). Pathways are sorted according to a *p* value (threshold 0.05) and corrected for a False Detection Rate (FDR). *p* value indicates extent of regulation of a particular pathway by specific microRNA(s). mirPath also provides the number and names of genes regulated by miRNAs in each KEGG pathway. We used mirPath-v1.0 and mirPath-v2.1 and, based on *p* value, the number of genes and the number of microRNAs involved, determined which pathways and diseases could be affected the most by the set of alcohol-regulated microRNAs.

### Wright-Giemsa Staining

We used Wright-Giemsa staining (Sigma, MO) to visualize cellular components of saliva following the standard protocol. Briefly, around 0.5 ml of saliva was transferred onto a glass slide without bubbles and spread out to a thin, even layer using the edge of another glass slide. After air-drying for several minutes, the slide was stained with 1–2 ml of the Wright-Giemsa stain for two minutes and rinsed with deionized water. Excess water was blotted and the slide was air-dried. Images of stained cells were captured using a brightfield mode on the Olympus FSX-100 microscope (Olympus, PA).

### Cell Sorting and Flow Cytometry

Saliva was diluted 1:1 with magnetic affinity cell sorting (MACS) running buffer and filtered through a 5 µM membrane (Millipore, MA) by centrifugation at room temperature for 30 min at 300 rpm. The cells were collected from the membrane using FcR Blocking Reagent (Miltenyi Biotec, CA) and labeled with anti-CD45-conjugated microbeads (Miltenyi Biotec). After 15 min of incubation at 4°C, cells were resuspended in 50 µL of MACS buffer and separated through an autoMACS cell separator. The unlabeled cells (negative fraction) passed through while the magnetically labeled cells (positive fraction) were retained within the column eluted later from the column and stained with FITC conjugated anti-CD-45 antibodies (Miltenyi Biotec). After a ten-minute incubation in the dark at 4°C, the cells were washed and resuspended in a 200 µL MACS buffer for flow cytometry. Flow cytometry was performed using an Influx™ Mariner 209s high speed flow cytometer equipped with 488 nm 200 W Argon blue excitation laser and 70 µm nozzle tip (BD Biosciences, CA). The software used to collect data was BD Software. For analysis, the trigger threshold was based on forward scatter (FSC). Unstained cells were identified by FSC and side scatter (SSC). Initially, cells were sorted out by their size to remove large buccal cells. Size gating was determined by geometric mean of FSC value relative to known fluorescent standard bead size (6–7 µm). Next, the remaining cells were characterized as human leukocytes based on their size and simultaneous detection of 520 nm wavelength emitted by FITC-conjugated anti-CD45 antibodies recognizing CD45 antigen, characteristic for white blood cells. Data were analyzed using FlowJo software (BD Biosciences) and shown as 3D density scatter plots and histogram plots. The 3D density scatter plots show two-parameter data (*X* vs. *Y* axes) with the different colors representing the frequency of events falling at each position. The histogram plots show single-parameter data (*X* axis) with *Y* axis representing the number of events (counts) for this parameter.

### Target Analysis

Four targets were selected using mirPath analysis of the KEGG (Kanehisa and Goto, 2000) adherens junction pathway in DIANA (Vlachos et al., 2012): catenin (CTNND1), a receptor tyrosine kinase (MET), a serine/threonine kinase (NLK) and a transcription factor (SNAi1). Targets were selected based upon the following criteria: 1) direct relevance to the pathway, 2) diverse cellular location and function, 3) prediction of being targeted by multiple miRNAs, 4) prediction of regulation by upregulated microRNAs only, 5) confirmation of possibility of regulation by microRNA *via* RNAhybrid (Rehmsmeier et al., 2004). Glyceraldehyde-3-phosphate dehydrogenase (GAPDH) was found to be a stable control under alcohol exposure in mammalian cells and was used as a reference (Maran et al., 2004; Rehmsmeier et al., 2004; Mason et al., 2012). Expression of each target was measured in at least 3 control and 3 alcohol samples.

Since each of these targets has several different alternative splice variants, based on bioinformatic and biological evidence in KEGG, NCBI and Ensembl, we designed qPCR primers and probes against the following transcripts: MET—ENST00000318493 and ENST00000397752, NLK—ENST00000407008 (Supplementary Table S1) using PrimerQuest software (IDT). PrimeTime primer and probe sets were selected to operate under the standard conditions for Taqman qRT-PCR assay, using 6-carboxyfluorescein (6-FAM) as the reporter. For detection of CTNND1, SNAi1 and GAPDH we used validated, pre-designed Taqman gene expression assays (Life Technologies)—Supplementary Table S1.

RNAhybrid (Rehmsmeier et al., 2004) was used to examine binding sites in the 3′UTRs of these transcripts as previously (Pietrzykowski et al., 2008). 3′UTRs of each transcript were obtained from Ensembl. Settings adjusted included: hits per target (10), energy cutoff (−19 kcal/mol), and helix constraint (2–8). Together these settings displayed only hits of −19 kcal/mol or lower, which had perfect matches to at least the seed region with a strong likelihood of being valid binding sites (Supplementary Figure S1).

### Quantitative Real-Time PCR Analysis of Individual miRNA Targets

Reverse Transcription (RT) was conducted using the SuperScript VILO cDNA synthesis kit (Life Technologies) according to manufacturer’s suggestions to create total cDNA from total RNA samples. Samples examined included 4 controls and 4 alcohol abusers for each target. Between 400ng and 1ug total RNA was used to generate cDNA for each sample. The thermocycler program used was 25°C for 10′ followed by 42°C for 120 min, 85°C for 5′, 4°C ∞. An extended time at 42°C was used as recommended in the protocol, to generate a higher quantity of template.

qPCR was conducted using the Taqman gene expression protocol and supplies on an ABI Step-one Plus machine (Life Technologies) using standard conditions and FAM as a reporter. Data was collected on the 60°C step at each cycle. No RT (no reverse transcriptase at RT step) and No template (No DNA added at qPCR step) were negative controls used in each run. The ΔΔCt method of analysis, comparing the results against GAPDH was used. Samples were internally compared against their own GAPDH, to result in accurate relative quantification.

### Statistical Analysis

GraphPad (v6.0a) for Mac (GraphPad Software Inc., La Jolla, CA) and R package were used for analysis. The Mann-Whitney *U* test was performed to detect differentially expressed miRNAs between groups (alcohol abusers vs. non-abusing controls). Pearson correlation with two-tailed *p* values was used to examine correlation between qPCR results and the corresponding array results, as well as correlation between microRNA expression and the amount of consumed alcohol. Correlations were expressed as a correlation coefficient (r) with *p* < 0.05 set as significant.

Receiver Operating Characteristic (ROC) Curve analysis was performed to determine sensitivity (true positive results) and specificity (true negative results) of the panel of 38 microRNAs, which expression was significantly changed in alcohol abusers compared to the control group, as a biomarker screen. The area under the ROC curve (AUC) quantifies the overall ability of the test to discriminate between individuals with the disease (i.e., alcohol abusers) and without the disease (i.e., controls). AUC values fall between 0.5 (no discrimination between two groups) and 1.0 (a perfect test with zero false positives and zero false negatives) with *p* value set at 0.05 indicating the significance of the test. ROC was used to compare the alcohol-specific microRNA panel with the control group, as well as each individual subject with the alcohol-specific microRNA panel to determine false positives and false negatives.

Principal Component Analysis (PCA) was performed using R package (Venables and Ripley, 2002), specifically “prcomp” function in R package “stats” (1) on the 38 miRNA expression dataset. Data were centered and scaled $\left[x/\left(x_{max}-x_{min}\right)\right]$ per variable before analysis. Data are shown as a 3D plot.

## Results

### Characterization of Patients

We wanted to establish whether profiling of microRNA in saliva could be a useful biomarker tool in distinguishing heavy alcohol drinkers from sporadic alcohol users. Thirty-eight patients at the primary health care center located in Trenton, NJ were considered for this study based on their chronic alcohol abuse or its lack. The characteristics of patients are summarized in Table 1. The alcohol-abusing patients were of both genders (82% males, 18% females), mixed ethnic backgrounds, and an average age of 47 years (+/− 7 years). The patients had been drinking on a daily basis for at least 10 years before admission with the average drinking period of 21 years. Their current alcohol intake is shown in Table 1. The non-abusing controls are shown in the lower part of Table 1. They also represent both genders and mixed ethnicity, with an average age of 40 years (+/−5 years). In contrast to the alcohol-abusing group, the control patients were drinking sporadically, no more than 20 g alcohol per drinking session.

**TABLE 1:** Characterization of patients and their drinking behavior. On a few occasions patients did not provide exact number of years drinking (designated by “many*”), however the duration was always more than 10 years.

#	Age (years)	Gender	Ethnicity	Drinking period (years)	Drinking pattern	Alcohol intake (gEtOH/24 h)
1	39	M	Hispanic	20	Daily	137
2	56	M	African Am	20	Daily	82
3	44	M	Caucasian	15	Daily	82
4	55	M	Caucasian	25	Daily	164
5	40	M	African Am	15	Daily	137
6	47	F	African Am	20	Daily	96
7	61	M	Caucasian	45	Daily	274
8	45	F	Caucasian	28	Daily	110
9	44	M	Caucasian	many*	Daily	82
10	44	M	Hispanic	10	Daily	1,096
11	49	M	Asia/India	20	Daily	55
12	50	F	African Am	35	Daily	205
13	55	M	Caucasian	10	Daily	55
14	35	M	Hispanic	many*	Daily	82
15	56	M	Caucasian	20	Daily	96
16	41	F	Caucasian	23	Daily	55
17	52	M	Caucasian	35	Daily	164
18	49	M	African Am	30	Daily	110
19	46	M	Caucasian	many*	Daily	466
20	46	M	Hispanic	10	Daily	274
21	33	M	Hispanic	15	Daily	1,096
22	45	M	Caucasian	10	Daily	137
Controls						
1	35	M	Hispanic	0	Occasionally	13
2	40	M	Caucasian	0	Occasionally	20
3	41	F	Caucasian	0	Occasionally	20
4	45	M	Caucasian	0	Occasionally	13
5	49	M	African Am	0	Occasionally	20
6	46	M	African Am	0	Occasionally	20
7	42	M	Caucasian	0	Occasionally	20
8	42	M	Caucasian	0	Occasionally	20
9	38	M	Caucasian	0	Occasionally	7
10	32	M	Caucasian	0	Occasionally	13
11	35	M	Caucasian	0	Occasionally	20
12	39	M	Caucasian	0	Occasionally	7
13	43	M	Caucasian	0	Occasionally	20
14	44	M	African Am	0	Occasionally	13
15	34	F	Caucasian	0	Occasionally	20

### Changes in microRNA Expression in Saliva of Alcohol Abusers

Human-specific, low-density microRNA microarrays (v3.0, Applied Biosciences) were used to measure microRNA expression profiles in the saliva of alcohol non-abusers and abusers. First, we compared microRNA profiling results of our control group with microRNA profiling in saliva of healthy controls of other studies. U6 was used as a reference microRNA.

**FIGURE 1:**
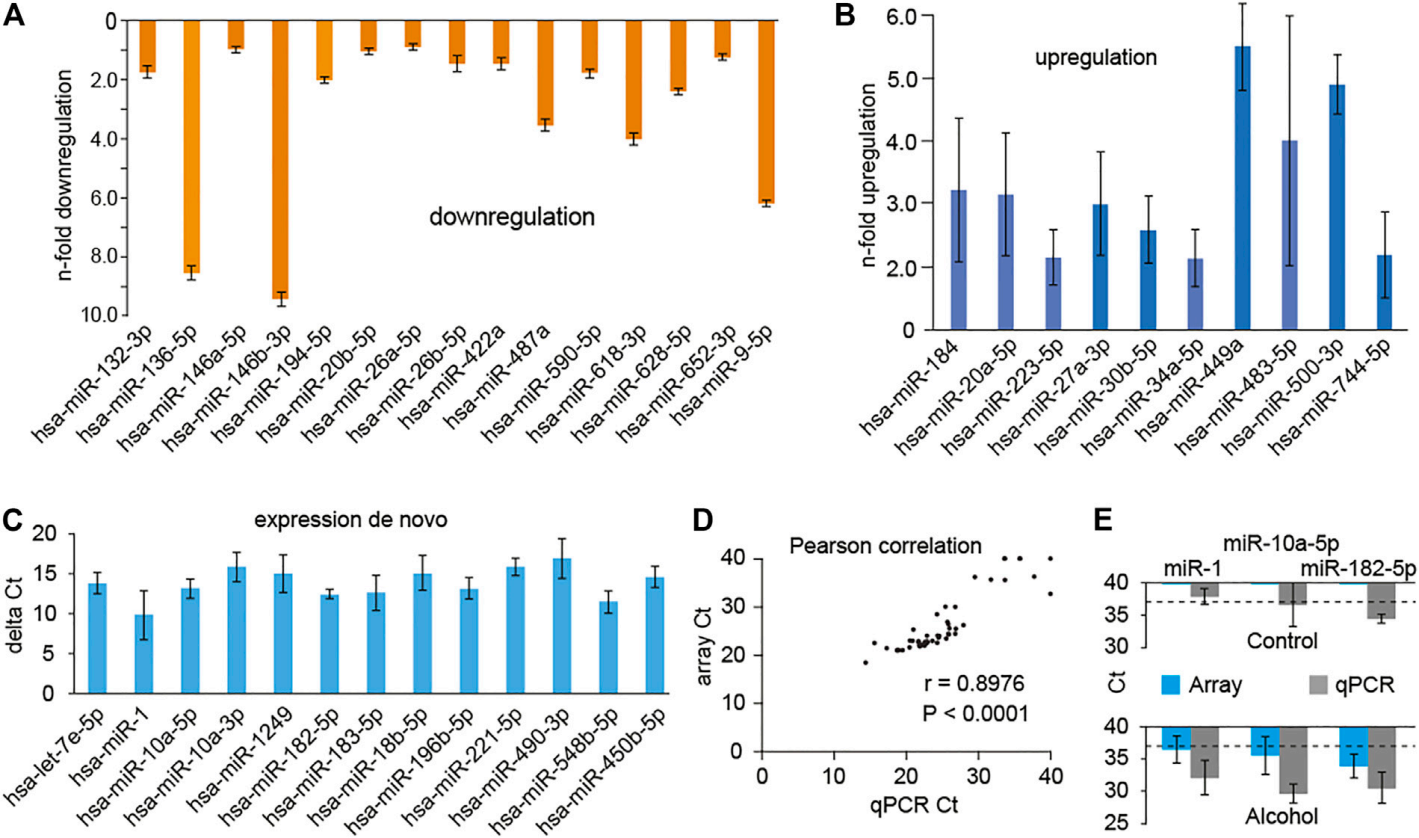
microRNA species, for which expression is significantly changed in the saliva of alcohol abusers (*n* = 22) compared to non-abusing controls (*n* = 15). **(A)** microRNAs downregulated compared to the control group. Expression levels are expressed as n-fold change. **(B)** microRNAs upregulated compared to the control group. Expression levels are expressed as n-fold change. **(C)** microRNA species upregulated *de novo*, meaning these microRNAs were undetected in the control group, but present in the alcohol-abusing group. Lack of expression in controls prohibits comparison of expression levels of these microRNAs between both groups, therefore results are presented as ΔCt of the alcohol abuser group. **(D)** High (*r* = 0.8976) correlation between microRNA microarray results and individual microRNA quantitative PCR (qPCR) results. **(E)** Validation of *de novo* upregulation of microRNAs in alcohol abusers. Representative microRNAs indicate borderline expression levels in controls, while detectable in alcohol abusers by both, arrays and qPCR. Note: microRNAs are named according to conventional nomenclature in which mature microRNAs coming from the complementary strands of a precursor are called miR-xx-5p and -3p, respectively, not miR-xx and miR-xx*. Significance determined using Mann-Whitney *U* test with *p* < 0.05. Bars represent the mean±SEM (Standard Error of the Mean). U6 was used as a reference.

Comparison of microRNA expression levels between alcohol abusers and controls revealed 38 significantly (*p* < 0.05) changed microRNA species, which fell into three distinct groups (Figure 1): 1) microRNAs downregulated in alcohol abusers compared to non-abusers, 2) microRNAs upregulated in abusers, and 3) microRNAs undetected in non-abusers but present in abusers (expression *de novo*).

The downregulated group consists of 15 microRNAs (Figure 1A): miR-132-3p, miR-136-5p, miR-146a-5p, miR-146b-3p, miR-194-5p, miR-20b-5p, miR-26a-5p, miR-26b-5p, miR-422a, miR-487a, miR-590-5p, miR-618-3p, miR-628-5p, miR-652-3p, and miR-9-5p. The downregulation ranged from 1 to almost 10-fold. The upregulated group consisted of 10 microRNAs (Figure 1B): miR-184, miR-20a-5p, miR-223-5p, miR-27a-3p, miR-30b-5p, miR-34a-5p, miR-449a, miR-483-5p, miR-500-3p and miR-744-5p. The range of upregulation was from two to almost six-fold. The expression *de novo* group is shown in Figure 1C. Since these microRNAs were undetected in the controls, their expression level cannot be compared to the control level and expressed as a fold change. Therefore, we show expression of these microRNAs as ΔC_t_, where values of expression have been normalized to a housekeeping microRNA (U6). The upregulated *de novo* group consisted of 13 microRNAs (Figure 1C): let-7e-5p, miR-1, miR-10a-5p, miR-10-3p, miR-1249, miR-182-5p, miR-183-5p, miR-18b-5p, miR-196b-5p, miR-221-5p, miR-490-3p, miR-548-5p and miR-450b-5p.

### Microarray Validation by Real-Time RT-PCR Analysis

To validate results from microRNA microarray analysis, we performed qRT-PCR. We chose ten microRNAs from each category and measured their expression in both groups: non-abusers and abusers. We also included microRNAs for which expression didn’t change or was undetectable in both groups. The miRNAs examined include hsa-miR-1, -10a-5p, -182-5p, -26a-5p, -27a-3p, -20a-5p, -29c-3p, -106a-5p, -9-5p, -618 and miR-U6 as a reference microRNA. All together we performed 60 individual qRT-PCR reactions in triplicate to validate microarray results. Overall correlation analysis indicated a high level of agreement between array and qRT-PCR data (*r* = 0.8976, *p* < 0.0001, Figure 1D, Supplementary Table S2) validating the microRNA microarray results.

Absence of microRNAs in the control subjects in the expression *de novo* group could be due to the low detection limit of microarrays. qRT-PCR is a more sensitive method able to detect a single copy of microRNA. Therefore, we used qRT-PCR to further test expression levels of some of the microRNAs in the expression *de novo* group using the same samples used for microarrays. Representative results are shown in Figure 1E. The qRT-PCR results confirmed that the level of expression of microRNAs in the control subjects of the expression *de novo* group is near or below detection limit, suggesting their physiological irrelevance in non-abusers. Furthermore, the qRT-PCR confirmed significant upregulation of these microRNAs in the abusers. Together, these results show significant deregulation of specific miRNAs in saliva of alcohol abusers, validating consideration of the use of microRNA profiling in the development of biomarkers of excessive alcohol consumption.

### Functional Analysis

To investigate the functional significance of the combinatorial changes in the microRNA expression in alcohol abusers we performed biological pathway analysis using DIANA mirPath software and KEGG database combined with biological measure of key elements in one of these pathways. Top five biological pathways affected the most are presented in Table 2 including number of genes in each pathway targeted by microRNAs, as well as most relevant upregulated and downregulated microRNA species.

**TABLE 2:** Top five biological pathways, which could be affected by an overall change in microRNAs expression in patients chronically abusing alcohol. Analysis was performed using DIANA mirPath and KEGG software with FDR correction and *p* value threshold of 0.05. Blue color depicts upregulated miRNA, orange—downregulated miRNA.

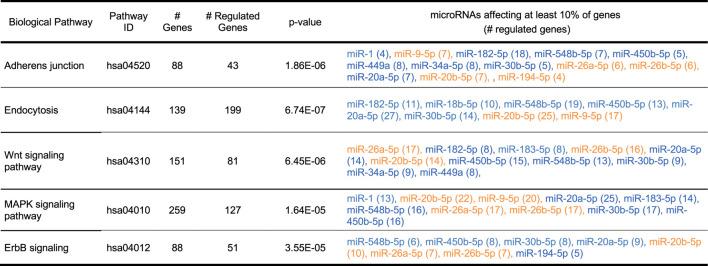

Overall, the impact of upregulated microRNAs seems to be larger than downregulated microRNAs (Table 2, right column). As the primary role of microRNA is suppression of gene expression (Miranda, 2014), it is permissible to assume that the increased levels of microRNAs would likely cause overall downregulation of their targets and impairment of function of pathways, to which the targets belong. Thus, this bioinformatic analysis suggests that several crucial cellular pathways could be affected by changes in microRNA expression in alcohol abusers *via* suppression of some key elements of these pathways.

**FIGURE 2:**
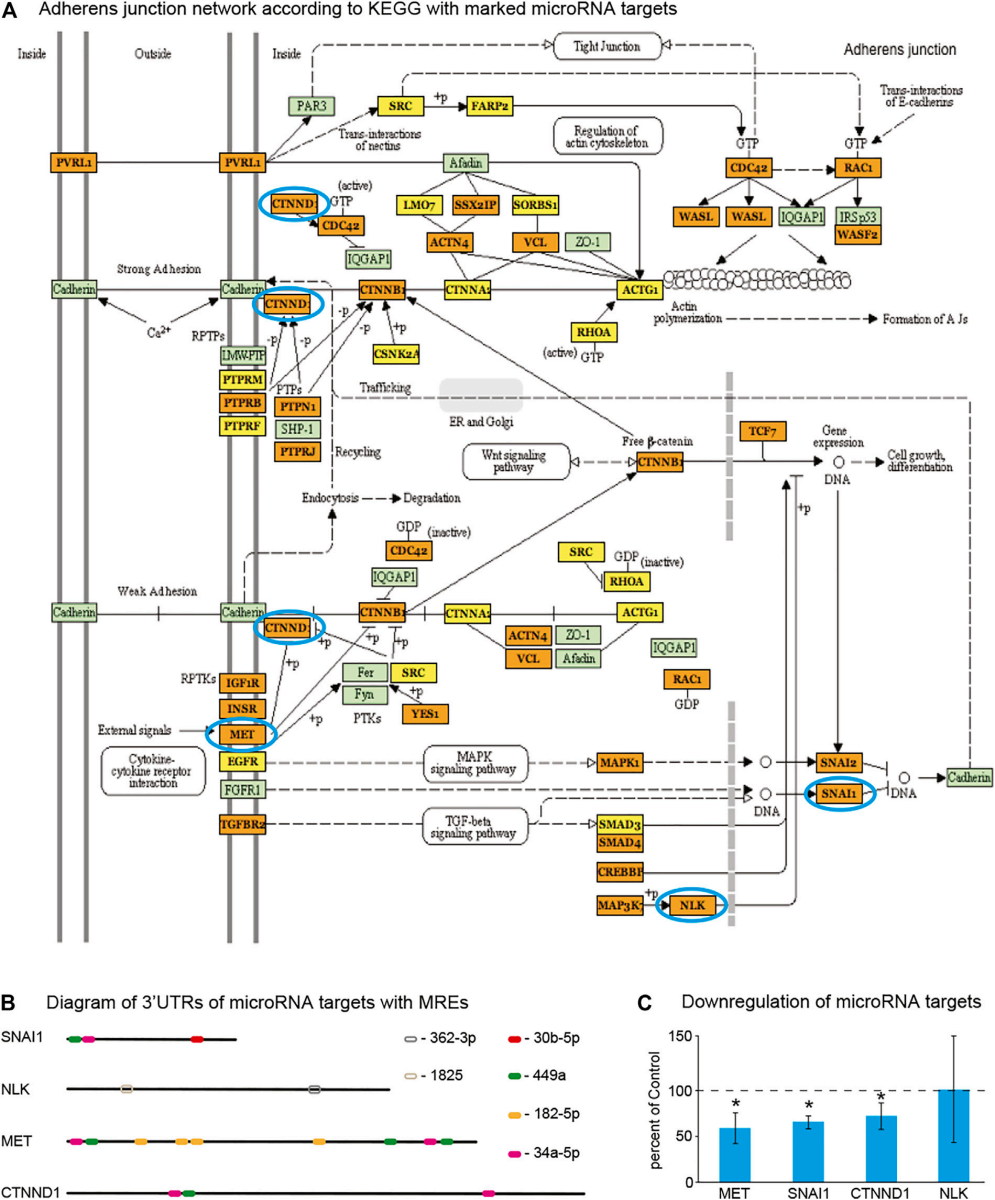
A wiring diagram showing potential regulation of adherens junction pathway by microRNAs relevant in alcohol abuse. **(A)** The diagram shows relationships of several gene products important for the proper function of the adherens junction. It also shows links to other pathways, some of them potentially regulated by alcohol. Gene products in yellow boxes are affected by one microRNA, gene products in orange boxes are affected by more than one microRNA. Gene products in green boxes are unaffected. Gene products in blue circles were tested by qPCR. The wiring diagram was created using KEGG (Kyoto Encyclopedia of Genes and Genomes) and DIANA-mirPath software with FDR correction and *p*-value threshold of 0.05. **(B)** Diagram of 3′UTRs of four genes [gene products in blue circles in **(A)**] targeted by several alcohol-regulated microRNAs (see the legend) showing relative positions of individual microRNA responsive elements (MREs). Empty circles indicate hybridization energy between a microRNA and its target above −20 kcal/mol. **(C)** Decreased expression levels of four microRNA targets marked in **(A)** and depicted in **(B)** in alcohol samples. GABDH was used as a reference. *N* ≥ 3. * - *p* < 0.05.

One of the top “hits” was adherens junction, which is a contact point between various types of cells, important in maintaining cellular function and tissue integrity. Indeed, miRNAs have been found to be crucial for the regulation and pathology of adherens junction (Cichon et al., 2014). Targeting of elements of the adherens junction pathway by alcohol-regulated microRNAs is shown as a wiring diagram in Figure 2A. Malfunction of adherens junctions has been indicated in the pathologies of the immune system (Wang et al., 2011; Kaymak et al., 2021) and the development of cancer (Vasioukhin, 2012; Bischoff et al., 2020). Since these diseases are aggravated by alcohol consumption (Sambuy, 2009; Meadows and Zhang, 2015), we decided to explore effects of microRNA de-regulation on the adherens junction pathway elements.

We focused on four targets located in different cellular compartments: 1) catenin (CTNND1)—a cytoplasmic protein associated with cadherin, plasmalemmal adherens junction receptor, 2) a plasma membrane receptor tyrosine kinase (MET), 3) a cytoplasmic kinase (NLK) and 4) a transcription factor (SNAi1) located in the cell nucleus (Figure 2A, blue circles). According to mirPath, transcripts of each of these genes were targeted by multiple microRNAs (Figure 2B), all of them upregulated. Since microRNAs work mainly as suppressors of gene expression, we assumed to observe an inverse correlation between miRNA expression changes and changes in the steady-state levels of their predicted targets. This was true for three of these targets: MET, SNAi1 and CTNND1 (Figure 2C) as determined by qPCR. However, the expression of the fourth tested target, NLK was unchanged (Figure 2C). In all cases GAPDH was used as a reference. Suppressive effect of microRNA on its mRNA target depends on the strength of thermodynamic interaction between both molecules. Closer examination of the strength of microRNA:mRNA target interaction by RNAhybrid (Rehmsmeier et al., 2004; Krüger and Rehmsmeier, 2006) revealed that the minimum free energy hybridization for any relevant microRNA with the NLK 3′UTR was above −20 kcal/mol (Supplementary Figure S1) suggesting a weak interaction (marked on Figure 2B as open ovals). In contrast, the minimum free energy of hybridization of all other downregulated targets with their microRNAs was below −20 kcal/mol (Supplementary Figure S1) indicating stronger interactions (Figure 2B—full ovals).

Altogether, modulation of cellular adherens junction pathway by alcohol *via* microRNA could have severe consequences on cell adherens by decreasing expression of important elements of the adherens junction pathway. Wiring diagrams of other pathways are shown in the Supplementary Figure S2.

### Cellular Content of Saliva

**FIGURE 3:**
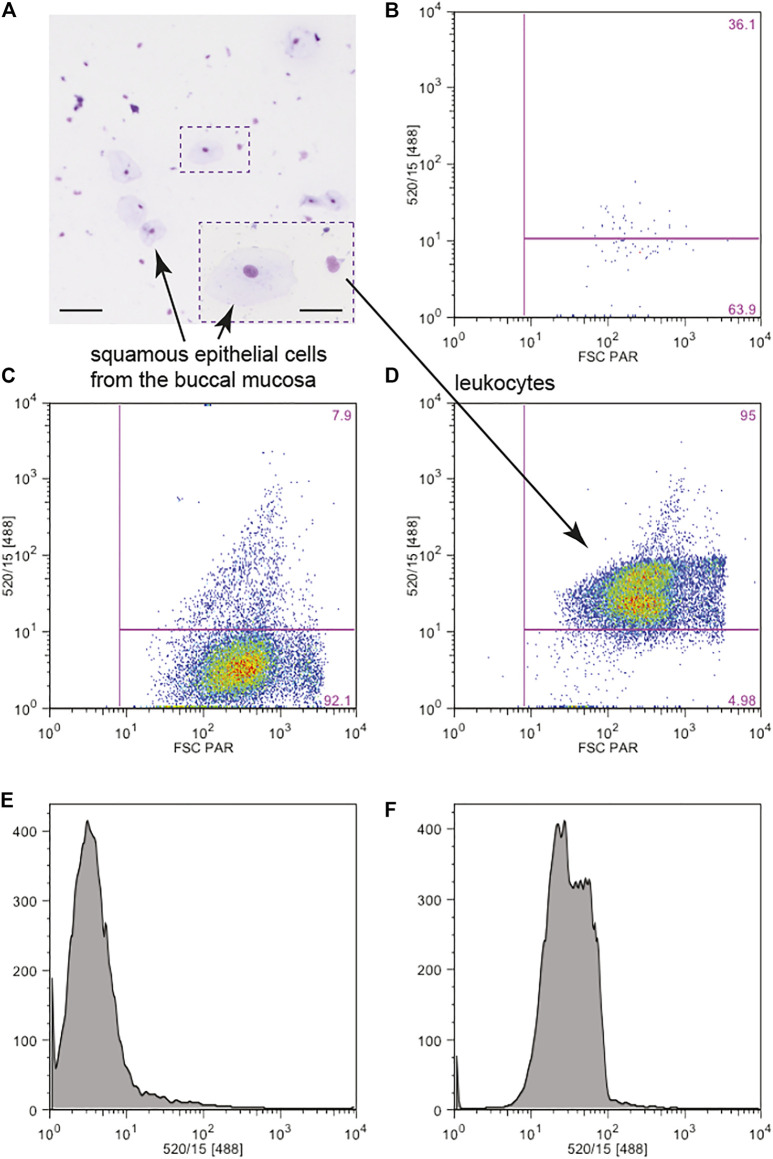
Saliva contains epithelial buccal cells and leukocytes. **(A)** Wright-Giemsa staining of saliva indicates the presence of large buccal cells as well as smaller cells resembling leukocytes. The rectangular area demarcated by the dotted line is enlarged in the right, bottom corner. Lower magnification scale bar—100 um, higher magnification scale bar—30 um. Cell sorting and flow cytometry showed the presence of leukocytes in saliva. **(B)** The unlabeled cells were not detected. **(C)** A representative 3D density scatter plot shows that most of the cells, when unlabeled, clustered into a homogeneous group in one gating window below the fluorescent threshold based on size representing leukocytes. **(D)** Upon labeling with fluorescent CD45-specific antibodies almost all of cells from **(B)** shifted above the threshold and accumulated in one gating window indicating that these cells are human leukocytes. **(E)** A representative histogram showing the homogeneity of unlabeled cells based on size. **(F)** Upon labeling with fluorescent CD45-specific antibodies almost all of cells from **(E)** shifted above the threshold indicating that these cells are human leukocytes. Red lines in **(B–D)** delineate gating windows. FSC—forward scatter, 520/488—fluorescent emission/excitation wavelengths.

Use of microRNA target prediction programs allowed us to understand better the impact of chronic alcohol abuse on cellular processes. To determine the main cellular source of human microRNA in the samples, we performed Giemsa staining (Figure 3A). We observed the presence of large cells of irregular shape with abundant cytoplasm—typical morphological features of squamous epithelial buccal mucosa cells (cheek cells, average size 50–70 µm diameter). We also detected much smaller cells (average size 9.4 µm diameter) with large nuclei and a rim of cytoplasm resembling leukocytes (Figure 3A). Indeed, immune cells constantly migrate to the oral cavity as the first line of defense against pathogens and they can be present in saliva (Schiott and Loe, 1970). To further determine the phenotype of these cells we performed flow cytometry first filtering out large cheek cells based on their size and then harvesting remaining cells using antibodies against CD45 (leukocyte common antigen) specific for human leukocytes. There were almost no cells left after filtering and harvesting (Figure 3B). In flow cytometry forward scatter (FSC) intensity is proportional to the diameter of the cell, therefore FSC signal can be used to discriminate cells by size. On FSC/Fluorescence 3D density scatter plot of unlabeled cells we observed specific clustering of the vast majority (92.1%) of the cells by macrophage-relevant size in one gating window, below the fluorescence threshold (Figure 3C). These cells, when labeled for presence of CD45 antigen, shifted above the fluorescence threshold and accumulated mostly (95%) as a single cluster of the cells of the same size as in (b) in one gating window of a scatter plot (Figure 3D). Homogeneity of cells and their specificity of expressing CD45 antigen can be also seen as a single peak on histograms, and the shift from below to above the threshold, as shown in Figures 3E,F, respectively. The shift of almost all cells from one gating window to another (from Figures 3C,D, and from Figures 3E,F), based on presence of CD45 antigen, indicated the presence of human leukocytes in saliva. Together, morphological and flow cytometry data indicate that the two major cell populations in saliva are squamous epithelial buccal mucosa cells and leukocytes.

### microRNA Profiling Supports Alcohol Contribution to Carcinogenesis

**FIGURE 4:**
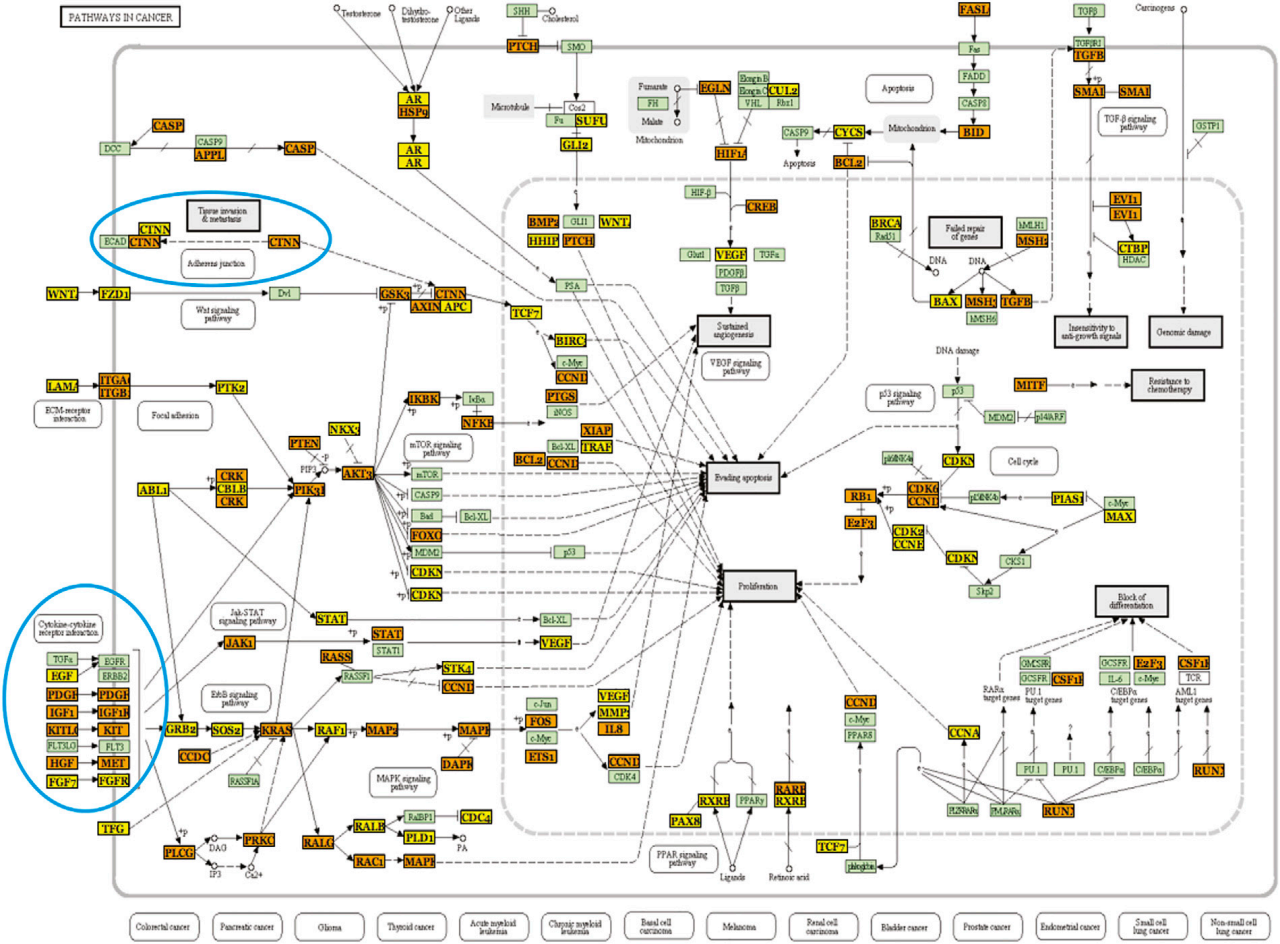
A wiring diagram showing pathways in cancer and their regulation by microRNAs relevant in alcohol abuse. The diagram shows a network of several pathways important in the development of cancer. Gene products in yellow boxes are affected by one microRNA, gene products in orange boxes are affected by more than one microRNA. Gene products in green boxes are unaffected. Elements of pathways in blue circles were tested by qPCR (Figure 2). Wiring diagrams of specific cancers are available in the Supplementary Materials. The wiring diagram was created using KEGG (Kyoto Encyclopedia of Genes and Genomes) and DIANA-mirPath software with FDR correction and *p*-value threshold of 0.05.

Each time alcohol is consumed, it travels throughout the body and can affect other cell types in addition to cells present in the oral cavity. Thus, changes in microRNA profiles observed in cells present in the oral cavity could potentially provide a window into possible changes in microRNA expression in other cell types. We have used mirPath software and KEGG database to determine the potential contribution of alcohol-sensitive microRNAs to pathogenesis of diseases. The top hits of our analysis are various types of cancer, all originating mainly from epithelial cells (Table 3). Summary of the potential regulation of cellular pathways in carcinogenesis by alcohol-sensitive microRNAs is shown as a wiring diagram in Figure 4 and in Supplementary Figure S3. Blue circles on Figure 4 depict cellular processes abnormal in cancer, which expression of some elements has been indicated to be aberrant by the current study. Interestingly, some of alcohol-regulated microRNAs suggested to be involved in pathogenesis of cancer, are also regulators of inflammatory responses, regardless of the cancer type (Table 3, right column), corroborated studies establishing a link between inflammation and cancer (Miranda, 2014). In this study, a group of alcohol-regulated, inflammatory microRNAs involved in all of these types of cancers include six microRNAs: miR-9-5p, miR-20a-5p, miR-20b-5p, miR-27a-3p, miR-30b-5p, and miR-182-5p.

**TABLE 3:** The overall change in microRNAs expression by alcohol abuse could lead to several cancers of epithelial origin. Analysis was performed using DIANA mirPath and KEGG software with FDR correction and a *p*-value threshold of 0.05. The top six cancers are shown. Inflammatory microRNAs are marked red.

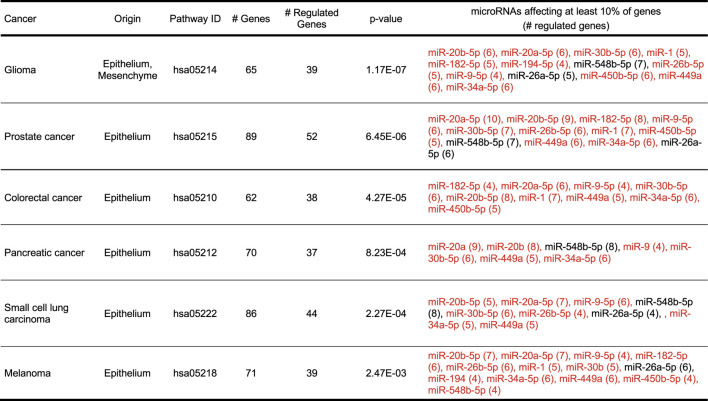

### microRNAs as Potential Biomarkers of Alcohol Consumption

**FIGURE 5:**
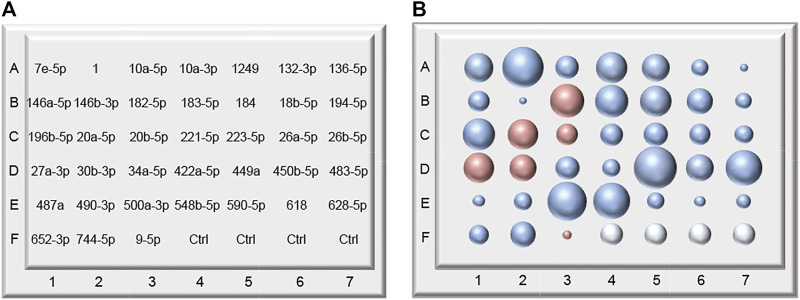
A hypothetical model of an alcohol abuse microRNA biomarker chip. **(A)** The chip contains a panel of 38 microRNAs characteristic of alcohol abuse. Four spots are designated to controls (bottom, right corner). **(B)** Results characteristic for alcohol abuse. The size of a sphere corresponds to the change in microRNA expression. Upregulated microRNAs are shown as blue spheres larger than controls, downregulated spheres are shown as spheres smaller than controls. The red color depicts inflammatory microRNAs.

Our study indicates that all together 38 microRNAs are differentially expressed in a group of heavy alcohol consumers compared to the control group (Figure 5). We attempted to measure the correlation between change in the expression level of individual microRNAs and the amount of alcohol consumed. We observed that three microRNAs expressed moderate to weak correlation: miR-196b-5p (*r* = −0.6672, *p* < 0.0127), miR-26b (*r* = −0.4273, *p* < 0.0473) and miR-223 (*r* = −0.4290, *p* < 0.0463).

**FIGURE 6:**
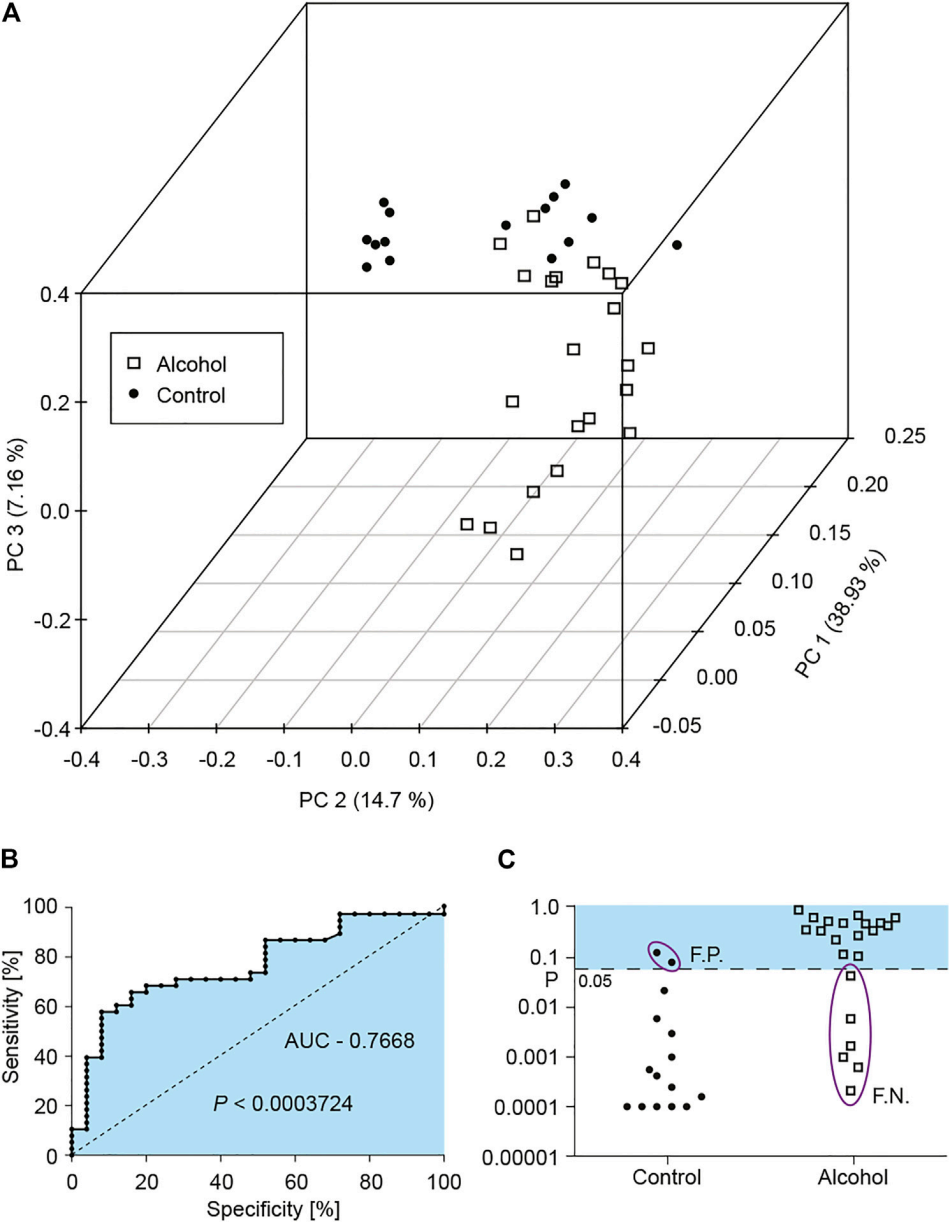
Efficacy of the current microRNA panel to segregate alcohol abusers and non-abusing controls. **(A)** 3D graph of Principal Component Analysis (PCA, PC1—38.93%, PC2—14.7%, and PC3—7.16% of the total variance) shows that most alcohol and control individuals can be classified into separate clouds with some overlap. **(B)** A Receiver Operating Curve (ROC) of alcohol-regulated microRNA panel indicates the ability of the panel to distinguish between the alcohol group and the control group: the area under the ROC curve (AUC, blue) is significantly above random sampling: 0.7668 (*p* < 0.0003724). **(C)** The fit of each individual to an overall alcohol-regulated microRNA panel is shown as a *p*-value of departure of individual ROC curve from the panel’s ROC with *p* = 0.05 used as a cut-off for significance (dotted line). If *p* ≥ 0.05 the microRNA panel determines the individual as an alcohol abuser, if *p* < 0.05 the microRNA panel classifies the individual as a non-abuser. False positives and false negative results are circled.

Next, we wondered whether the microRNA panel as a whole could adequately segregate alcohol abusers from control non-abusers. First, we used the Principal Component Analysis (PCA), because PCA has been widely used to reduce high-dimensional data and to determine the similarities and dissimilarities of biological samples without losing too much information (van der Werf et al., 2006; de Haan et al., 2007; Simmons et al., 2015). In this study, PCA was applied to the 38 miRNAs expression data of 22 alcohol and 15 control individuals. The three PCs account for around 61% of total variance: PC1 explains 38.93% of the total variance in this dataset, PC2 explains 14.7% of the variance, and PC3 explains 7.16% of the variance (Figure 6A). The 3D plot of three principle components showed that most of alcohol and control individuals could be segregated into separate clouds with some overlap (Figure 6A).

Next, we used Receiver Operating Characteristic (ROC) Curve analysis to determine sensitivity and specificity of the microRNA panel, and to calculate false positive and false negative values. The area under the ROC curve (AUC) quantifies the overall ability of the test to discriminate between individuals with the disease (*i.e.,* alcohol abusers) and without the disease (*i.e.,* controls). An AUC of 1 would indicate a perfect test of 100% sensitivity and 100% specificity, while an AUC value of 0.5 would show no discrimination between two groups (Figure 6B, dotted line). The ROC used to compare the alcohol-specific microRNA panel with the control group had the AUC value of 0.7668 significantly above random sampling (*p* < 0.0003). We next used the ROC analysis to determine how well each individual enrolled in the study (either control or alcohol abuser) would be classified to the alcohol group by the microRNA alcohol panel. Figure 6C shows the probability of each individual’s microRNA screen to differ from the microRNA alcohol panel. Two out of fifteen control individuals (13%) are false positives (Figure 6C, control, purple oval), while six out of twenty-two alcohol abusing individuals (27%) are false negatives (Figure 6C, alcohol, purple oval), showing rather encouraging results, and warranting pursuing further the use of microRNA profiling in saliva as a biomarker of alcohol abuse.

## Discussion

Alcohol Use Disorder (AUD) is a complex chronic disease affecting a large portion of our society. AUD is characterized by escalation of alcohol consumption over years, development of tolerance and addiction, and occurrence of relapse periods thwarting abstinence efforts. The pathological effects of alcohol on bodily organs vary, depending in part on the amount of consumed alcohol and the length of the excess of alcohol consumption. For example, chronic alcohol abuse is frequently associated with malfunction of the immune system, and can also lead to the development of several cancers (Meadows and Zhang, 2015).

Since AUD is a very serious societal issue, establishment of alcohol biomarkers is of great importance for clinical practice, public safety, criminal justice and research (Litten et al., 2010; Nanau and Neuman, 2015). A biomarker is defined as any substance present in an organism which can be measured and used to indicate specific functions of the body’s systems in health and disease. Biomarkers can have an important diagnostic, prognostic and pharmacological value in the course of a development of an ailment. The field of biomarker development is rapidly growing (see Nanau and Neuman, 2015; Rosato et al., 2019). Development of biomarkers in AUD would help to determine an advancement of the disease, early detection of its complications, and/or efficacy of treatment.

Here, in our studies, we compared heavy, chronic abusers of alcohol with at least ten-year long history of alcohol abuse, with a matched non-alcohol abusing control group, to determine whether chronic excess of alcohol changes expression of miRNAs in saliva, and whether these changes have a potential to serve as a biomarker of alcohol abuse.

microRNAs are attractive biomarker candidates. Expression of microRNAs in saliva can change with a disease progression (Rosato et al., 2019; He et al., 2020), and they are more stable in saliva than mRNAs (Pritchard et al., 2012; Han et al., 2018) reducing variability and improving accuracy. Indeed, microRNAs in saliva have been used as or theorized as useful biomarkers in oral and pancreatobiliary tract cancer, as well as head and neck squamous cell carcinoma (He et al., 2020; Machida et al., 2016; Langevin et al., 2017). Simultaneous measurement of expression of several microRNAs (microRNA profiling) provides a more powerful diagnostic tool (Jones et al., 2012; Rosato et al., 2019; Mahnke et al., 2021), therefore we screened the expression of several hundred microRNAs to determine a unique microRNA signature of chronic alcohol exposure and its potential as a biomarker of alcohol abuse.

Due to our stringent inclusion/exclusion criteria the enrolled number of individuals (38) was rather small but sufficient to see significant differences between two studied groups. We excluded many individuals with a known history of using any drugs of abuse including nicotine, which is very frequently co-abused with alcohol. The main experimental variable between two groups studied here was alcohol consumption, and we feel confident that observed differences are most likely attributable to chronic alcohol exposure. Ten to fifteen samples have been shown through statistical analysis to be sufficient for the stable replication of findings in microarrays (Pavlidis et al., 2002), and to perform microRNA profiling in diagnosis of cancer (Jones et al., 2012), supporting the validity of our studies. Moreover, the observed 85.5% concordance with controls (*n* = 18) of Hanson et al. (2009), 91.3% concordance with controls (*n* = 12) of Park et al. (2009), and 100% concordance with controls (*n* = 20) of Patel et al. (2011) reports indicating that our control microRNA profiles can accurately represent microRNA expression in healthy individuals.

It is currently accepted that age, gender and ethnicity play a role in the development of AUD (Gupta et al., 2018; National Institutes of Health, 2021a) and in discovery of biomarkers (Tarallo et al., 2018; Rosato et al., 2019). However, the progress of human civilization now blurs ethnic, gender and age divisions, which thus become less and less relevant. Our data indicate that microRNA profiling in saliva could be a reliable biomarker of chronic alcohol consumption with rather strong predictive power, regardless of age, gender and ethnicity.

Saliva is a very promising source of biomarkers. Some commercially available kits (Genotek) ensure immediate stabilization of RNA after collection of saliva. The presence of bacteria in the oral cavity does not bias the results of microRNA profiling, as many oral bacteria are not disrupted during human microRNA isolation process due to thick bacterial walls, and the human microRNA microarrays are specific for human microRNAs. Moreover, the collected samples can be designated as non-biohazard material, since the samples are self- collected, immediately placed in sealed containers, and exposed to a SARS-CoV-2 virus inactivating agent, which causes a >99% reduction in infective virus (DNA Genotek, 2021). Nevertheless, we recommend taking further precautions during collection and shipping samples following local, national and international regulations, including the current edition of the International Air Transport Association (IATA) Dangerous Goods Regulations.

Continuation of this study with various AUD groups can likely help further hone the microRNA biomarker panels, as more miRNAs related to alcohol exposure are being identified in saliva (Rosato et al., 2019). microRNA biomarkers could be also combined with other, non-microRNA alcohol biomarkers. For example, alcohol metabolites are currently considered as alcohol exposure biomarkers. Two of the most promising alcohol metabolites biomarkers, are carbohydrate-deficient transferrin (CDT), and ethyl glucuronide (EtG) (Litten et al., 2010; Nanau and Neuman, 2015). Both are highly sensitive and specific to alcohol intake, however require collection of bio-hazardous blood, and the use of rather expensive detection techniques present only in specialized laboratories: high performance liquid chromatography (HPLC) and liquid chromatography-mass spectroscopy (LC-MS), respectively (Litten et al., 2010; Nanau and Neuman, 2015). microRNA detection on the other hand is based on PCR methodology, which became recently easily-available in many first-contact diagnostic centers due to the COVID-19 pandemic.

Genotyping can also complement the role of microRNA biomarkers in AUD. Characterization of an alcoholism-susceptible genotype can provide “static” information of potential vulnerability to the development of alcoholism, while microRNA profiling can permit “dynamic” monitoring of the disease status, escalation of alcohol consumption, development of tolerance, continuity of abstinence or occurrence of relapse.

Since microRNAs are very important regulators of gene expression, profiling of microRNA could also provide insight into molecular mechanisms of alcohol actions on cell biology. Thus, to understand biological consequences of microRNA dysregulation in saliva we first determined that the most prominent cellular source of miRNAs are buccal cells and leukocytes. Although we cannot exclude the contribution of exosomal microRNA to the profiling, as exosomal miRNAs are present in saliva (Hu et al., 2012; Wong 2015), many of them originate from oral cavity cells such as the salivary glands (Han et al., 2018; He et al., 2020), making them useful indicators of oral, if not general, health.

Analysis of miRNAs in relation to associated signaling pathways revealed insights into disease mechanisms. Almost all of the top cancers suggested by bioinformatic analysis in this study originate from epithelial tissues (Table 3). There is some evidence that alcohol could contribute to the pathogenesis of these cancers (Vagst et al., 2003; Gupta et al., 2010; Michaud et al., 2010; Fedirko et al., 2011; Morris et al., 2015). However, none of the enrolled patients in our study has been diagnosed yet with these cancers. This might be because the median age of diagnosis of these cancers falls into the sixth decade of life (Between 57 years old for glioma to 71 years old for pancreatic cancer) according to the National Cancer Institute (Howlander et al., 2012). It takes a few decades for a transformation of a normal cell into a detectable cancer according to the multistage clonal expansion carcinogenesis model (Luebeck et al., 2012). During this period cancer remains undiagnosed (Luebeck et al., 2012), though improvements to modeling continue to shave down the delay (Avanzini et al., 2020). Interestingly, chronic alcohol consumption can lower the age of diagnosis from five (colorectal cancer, Zisman et al., 2006) to ten years (pancreatic cancer, Anderson et al., 2012). It is tempting to speculate that changes in microRNA expression could be a very early indication of de-regulation of biological processes, which could potentially lead to cancer in later years, thus making microRNA profiling an early biomarker of cancer development.

Comparison between our work and a salivary biomarker study in the literature for AUD (Rosato et al., 2019) found hsa-miR-10a-5p and miR-27a-5p as significantly upregulated in both studies. Alterations in miR-10a expression have been important in multiple types of cancers, likely through its role in the regulation of p53 (Ovcharenko et al., 2011; Bryant et al., 2012; Vu et al., 2021), a highly conserved, critical tumor suppressor gene. As a result, miR-10a has proven to be an important cancer biomarker (Vu et al., 2021). Given that alcohol abuse elevates the risk of many cancers (Fidler, 2003; International Agency for Research on Cancer, 2010; Wong et al., 2011; Zakhari et al., 2011; Miranda, 2014), it seems plausible that the dysregulation of miR-10a in alcohol abusers likewise leads to dysregulation of the p53 network in AUD individuals, underscoring how alcohol may act to elevate cancer risk. Likewise, miR-27a plays a role in tumorigenesis in multiple cancers (Li et al., 2019; Zhang et al., 2019; Cui et al., 2020).

We used a blend of bioinformatics (DIANA-mirPath, RNAhybrid) and wet-lab experiments (qPCR) to predict global impact of alcohol-regulated microRNAs on biological pathways and cellular processes. As recently suggested, targeting of transcripts by microRNA remains the same in the presence of alcohol (Miranda, 2012). We determined that several biological pathways could be affected, and we focused on the adherens junction pathway because adherens junctions are key for cell-cell interactions, act as contact points between cells and maintain tissue integrity. Changes in adherens junctions can cause changes in gene expression affecting cell adhesion and movement (Meadows and Zhang, 2015). Importantly, alcohol, by impairing adherens junction pathway, disrupts endothelial integrity increasing probability of bacterial infections (Wood et al., 2013) and metastatic cancer (Xu et al., 2012; Madoz-Gúrpide et al., 2015). The essential element of the adherens junction is transplasmalemmal cadherin-catenin complex, responsible directly for cell adhesion. We observed that alcohol-regulated microRNAs, could contribute to alcohol impairment of the adherens junction by targeting this complex in at least two ways. We detected significant downregulation of catenin transcripts, as well as transcripts encoding SNAil1, a transcription factor controlling expression of cadherin. Both types of molecules were targeted by several upregulated microRNAs.

The most downregulated element of the adherens junction and a target of alcohol-upregulated microRNAs was MET, a receptor tyrosine kinase, which plays a key role in c-Met signal transduction pathway. Activation of MET leads to multiple, diverse biological effects, while its de-regulation contributes to tumor progression and metastasis (Ma et al., 2003; Puccini et al., 2019). In has been shown that alcohol decreases expression of catenin in human neuronal stem cells (Vangipuram and Lyman, 2012) as well as its function in human bone marrow (Yeh et al., 2008), however increases catenin expression in the superior frontal cortex of chronic alcoholics (Al-Housseini et al., 2008), and in the hippocampal neurons of rats (Velázquez-Marrero et al., 2016). MET expression is not affected by alcohol in Chang (HeLa) liver cells [Ohira et al., 1995; Hall review discusses that Chang liver cells are HeLa cells (Hall, 2017)]. This is the first report indicating downregulated expression of catenin, SNAIL1 and MET in heavy alcohol drinkers. Future studies will be of importance to determine the exact contribution of de-regulation of these molecules by alcohol-regulated microRNAs to the disruption of cell adherence or increased tumor metastasis.

Interestingly, downregulated catenin is also a part of the Wnt signaling pathway, which was another mirPath top “hit”. Other potentially de-regulated pathways included mitogen-activated protein kinase (MAPK), ErbB signaling and endocytosis. MAPK and ErbB signaling pathways are both part of the cellular responses to an array of stimuli, and are involved in transfer of signaling to the nucleus and regulation of gene expression in cellular proliferation, migration or differentiation. Endocytosis is a ubiquitous cellular mechanism allowing delivery of plasmalemmal elements and vesicular cargo into cellular milieu. Alcohol disrupts Wnt signaling (Lauing et al., 2012; Vangipuram and Lyman, 2012; Kapania et al., 2020), changes phosphorylation of p38 MAPK (Gu et al., 2013; Xu et al., 2016) and ErbB (Srivastava et al., 2011; Xu et al., 2016), and impairs endocytosis (Marin et al., 2010). Our results suggest that alcohol de-regulation of microRNAs could be, at least partially, involved in these perturbations.

Impairment of these pathways in epithelial cells can lead to important pathologies. Loss of adherence of the epithelial cells to the surrounding environment is a known step in the epithelial-mesenchymal transformation (EMT) towards malignancy. Indeed, chronic alcohol exposure increases occurrence of oral cancer (Pelucchi et al., 2011) originating from squamous epithelial buccal mucosa cells (Johnson et al., 2011; Leite et al., 2021).

Further functional studies are necessary to definitively indicate that de-regulation of biological pathways by alcohol is microRNA-dependent. Such studies should consider the validation of microRNA targets. We used several criteria to validate some of the microRNA targets in the adherens junction pathway. In one of the top pathways indicated by mirPath enrichment analysis, we have chosen several elements of the selected pathway based on their role in the pathway, cellular location, targeting by multiple microRNAs and regulation by microRNAs, which expression was changed in the same direction (all microRNA upregulated). Next, we determined the probability of microRNAs interaction with these targets by an independent program (RNAhybrid), considering presence of multiple splice variants and 3′UTR heterogeneity of selected targets. We observed that one of the targets indicated by mirPath had hybridization energy above −20 kcal/mol, and its levels were not changed as determined by qPCR. The other targets were significantly downregulated as predicted. These results indicate that some genes in mirPath-selected pathways can be false positive.

Proper function of leukocytes also depends on cell motility and adherence. Impairment of these cellular properties by alcohol-regulated microRNAs in leukocytes could be one factor contributing to the known immunosuppressive effects of alcohol (Cook, 1998; Szabo, 1999; Messignham et al., 2002). microRNA regulation of the immune system also plays important role in regulation of inflammatory networks (O'Connell et al., 2012; Morris et al., 2015; Ureña-Peralta et al., 2018). Alcohol is known for its pro-inflammatory actions. Moreover, carcinogenic effects of alcohol are also thought to take place *via* modulation of inflammatory responses (Seitz and Stickel, 2007; Qin et al., 2008; Morris et al., 2015). We observed that out of 38 signature microRNAs, seven are involved in inflammation: miR-20a-5p, miR-20b-5p, miR-27a-3p, miR-30b-5p, miR-182-5p, miR-183-5p, and miR-9-5p. Specifically, miR-27a-3p and miR-30b-5p expression changes as a response to an inflammatory stimulus (Chang et al., 2012; Graff et al., 2012), while expression of miR-182-5p is altered in steatohepatitis (Dolganiuc et al., 2009). More recently, miR-182-5p and 183-5p, in the miR-183 cluster, were found to be dysregulated in mice exposed to chronic alcohol exposure (Osterndorff-Kahanek et al., 2018; Ureña-Peralta et al., 2018), a particularly relevant model for AUD patients. Both miR-182-5p and miR-183-5p regulate neuroinflammatory pathways involved in Toll-like receptor 4 (TLR-4) signaling, critical in innate immunity (Ureña-Peralta et al., 2018). Interestingly, miR-27a has been recently found to be upregulated in blood plasma from AUD individuals, underscoring its utility in signaling alcohol abuse (Saha et al., 2016).

Let-7, a miRNA in our study, has also been observed in inflammatory processes to act *via* TLR-7 regulation in alcoholism (Coleman et al., 2017; Crews et al., 2017). miR-155 and -199, while not observed in our studies, were implicated in studies of rat liver cells and endothelial cells in humans in response to chronic alcohol insult, and lead to inflammation in cirrhosis through endothelin-1 (ET-1) and hypoxia-inducible factor-1α (HIF-1α) (Yeligar et al., 2009). In a recent report of miRNA in immune cells from chronic inflammatory states (Salvi et al., 2019), several of the miRNA (miR-20b, -26a, -223) we found to be differentially regulated in our alcohol abusing population were also found and observed to be regulated in the same direction as in chronic inflammatory states. We found that let-7e was also regulated in the same direction, though the same target was not predicted for let-7e, but instead for the closely related let-7d, which likely overlaps many of the same targets. Most of these (all except miR-26a) were predicted by TargetScan 8.0 (McGeary et al., 2019) to have potential targets the same as known targets of miRNAs regulated in chronic inflammatory states primarily in immune cells as reported in Salvi et al. (2019). This suggests that these miRNAs are playing a similar role in the pathology of inflammation seen in chronic alcohol abuse.

miR-9 is a particularly interesting microRNA involved in alcohol actions and inflammation (Miranda, 2014). We have previously reported that acute alcohol (15 min exposure) causes upregulation of miR-9 (current nomenclature: miR-9-5p) and the development of molecular tolerance in neurons (Pietrzykowski et al., 2008). Alcohol exposure in fetal rats has been shown to upregulate miR-9, leading to dysregulation of pituitary dopamine D2 receptors (Gangisetty et al., 2017). A report by Sathyan et al. (2007) showed downregulation of miR-9 upon 24 h alcohol exposure in neurospheres. Our current results indicate that, in chronic alcohol abuse, miR-9 levels are downregulated in leukocytes and/or epithelial cells. This follows a usual pattern seen with addiction and tolerance; that is, abuse leads to a change in expression of a gene, but repeated or chronic abuse leads to tolerance and expression change in the opposite direction as the body tries to maintain homeostasis in the face of repeated spikes of the offending drug (Volkow and Morales, 2015; Cahill et al., 2016). Bazzoni et al. (2009), describe an inflammatory role of miR-9 in leukocytes. Together, these findings indicate that alcohol-sensitive microRNAs, like miR-9, could have a drastically different response depending on the length or frequency of alcohol exposure. Moreover, understanding temporal regulation of miR-9 and other inflammatory microRNAs by alcohol is of great interest in AUD, which is a chronic disease with an important inflammatory component (Kelley and Dantzer, 2011; Ureña-Peralta et al., 2018).

AUD affects chronic inflammation, at least partially, *via* increasing oxidative stress (Bala and Szabo, 2012). Alcohol consumption and metabolism leads to Reactive Oxygen Species (ROS) production (Rao, 2008; Szabo and Bala, 2010; Bala and Szabo, 2012), instrumental to pathological developments in Alcoholic Liver Disease (ALD) (Keshavarzian et al., 2009; Szabo and Bala, 2010). Several miRNAs, miR-27a-3p, miR-34a, miR-223, found to be differentially expressed in alcohol abusers in our study, were found to be differentially regulated in other physiological studies of oxidative stress (Bala and Szabo, 2012; Klieser et al., 2019). In particular, macrophages may be regulated by miRNAs in response to oxidative stress through the NF-κB pathway (Thulasingam et al., 2011).

Exposure to alcohol affects several inflammatory cascades including production of pro-inflammatory cytokines by many cells throughout the body (Qin et al., 2008). miRNAs can regulate pro-inflammatory cytokine pathways in chronic alcohol abuse (Coleman et al., 2017; Crews et al., 2017; Salvi et al., 2019). Several reports of individual microRNAs present on our panel describe microRNA regulation of pro-inflammatory cytokines, which can both attenuate or enhance cytokine production or secretion. Selected representatives (one per each of our three distinct microRNA groups) of such regulation include: secretion of pro-inflammatory cytokines is decreased by miR-20b targeting VEGF (Song C. et al., 2012), miR-20a targeting ASK1 (Philippe et al., 2013) and TLR4 (Li et al., 2020), and miR-183 targeting TGFa (Tao et al., 2021). On the other hand, secretion of pro-inflammatory cytokines is increased by miR-132-3p targeting TRIB1 (Niespolo et al.,. 2020), miR-223 targeting IKKa (Valmiki et al., 2020) or miR-183-5p targeting PPP2CA (Guo et al., 2020). These few examples underscore the importance of performing a comprehensive screen of multiple microRNAs to better understand the functional outcomes of microRNA regulation of biological pathways.

Overall, our data help strengthen the pathogenic link between alcohol, inflammation and malignancies such as cancer, providing attractive plausible molecular mechanisms involving specific microRNAs. In view of the non-invasive nature of sample collection, suggestive involvement of de-regulated microRNAs in alcohol-related pathologies and potential use of these microRNAs as an alcohol biomarker, a continuation of this study on a larger group of patients is of great interest. Obtaining samples from patients at different stages of AUD could allow the establishment of fine-tuned correlation between disease states, specific microRNAs and their patterns of modulation. The current study helped to narrow down alcohol-relevant microRNAs in humans to 38 microRNA species using two end-point groups: sporadic drinkers (controls) and heavy drinkers. A power analysis is a possible method to reliably estimate the number of subjects (n) (Pavlidis, Li, and Noble, 2002; Ching et al., 2014). However, power analysis typically requires prior knowledge of the topic under bioinformatics investigation (Pavlidis, Li, and Noble, 2002). Our study, performed on two end-point groups, could provide this information and should aid in performing power analysis to define the number of subjects necessary to determine the usefulness of the microRNA panel as a biomarker in various alcohol-drinking groups. Our results suggest also that changes in the expressions of miRNAs in the saliva of alcohol abusers could be used to monitor the effectiveness of any therapeutic approaches, including medications and lifestyle.

In summary, this study underscores that measuring changes in the expression of microRNAs in saliva may have predictive value in determination of alcohol abuse and its consequences. We also demonstrate the potential feasibility and provide direction for establishing microRNA profiling in saliva as a sensitive and specific tool in determination of chronic, excessive alcohol consumption. Further studies are needed to establish microRNA profiling as a biomarker of various stages of the alcohol use disorders and its sequelae.

## Data Availability

The original contributions presented in the study are included in the article/Supplementary Material, further inquiries can be directed to the corresponding authors.
