# Supplementary material for: Non-Invasive microRNA Profiling in Saliva can Serve as a Biomarker of Alcohol Exposure and Its Effects in Humans
Source: Front Genet. 2022 Jan 20;12:804222. doi: 10.3389/fgene.2021.804222 (PMC8812725; doi:10.3389/fgene.2021.804222)
Supplement: Supplementary file 1 [file Table1.docx]

**Supplementary Table S1. Amplicons of four adherens junction targets:** MET, NLK, SNAi1, CTNND1 and GABDH. Custom-designed forward primer, reverse primer and the probe for each amplicon is shown except commercially available amplicons.

**Custom-made amplicons**

| **Primer** | **Sequence (5'->3')** | **5'**  **Modification** | **3’**  **Modification** | **Tm (°C)** |
| --- | --- | --- | --- | --- |
| MET  Forward | GGCCATCGATATTCTTTGCTCTT | None | None | 63 |
| MET  Reverse | ATTGGTCCGTGGCCTGT | None | None | 64 |
| MET  Probe | AGCATCAGAACCAGAGGCTTGGTC | 6-FAM | IBFQ | 68 |
| NLK  Forward | TTGCCTGCGACCAATCAT | None | None | 62 |
| NLK  Reverse | CATCACCATCACGGCTCTAAT | None | None | 62 |
| NLK  Probe | TAGGGCTTGATCATTAGGTGGCATGC | 6-FAM | IBFQ | 68 |

6-FAM - 6-Carboxyfluorescein; IBFQ - Iowa Black Fluorescent Quencher

**Commercial Taqman (ABI) amplicons**

| **Target** | **Source** | **Product Information** | |
| --- | --- | --- | --- |
| GAPDH | LTI | Cat # 4331182 | Item # Hs03929097_g1 |
| SNAi1 | LTI | Cat # 4453320 | Item # Hs00195591_m1 |
| CTNND1 | LTI | Cat # 4453320 | Item # Hs00931670_m1 |
