# Supplementary material for: Non-Invasive microRNA Profiling in Saliva can Serve as a Biomarker of Alcohol Exposure and Its Effects in Humans
Source: Front Genet. 2022 Jan 20;12:804222. doi: 10.3389/fgene.2021.804222 (PMC8812725; doi:10.3389/fgene.2021.804222)
Supplement: Supplementary file 2 [file Image2.pdf]

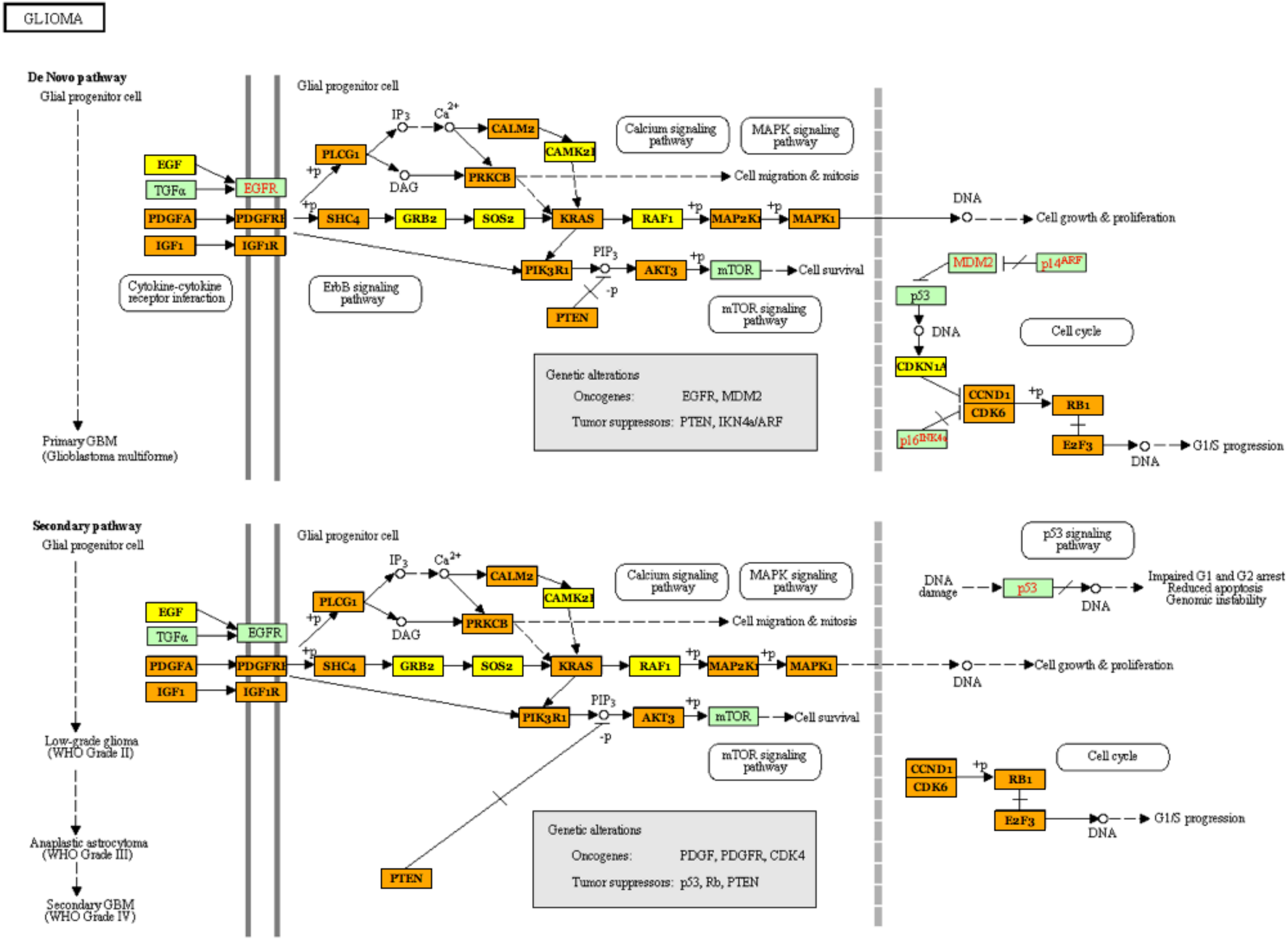
GLIOMA
De Novo pathway
Glial progenitor cell
Primary GBM
(Glioblastoma multiforme)
EGF
TGFα
PDGFA
IGF1
EGFR
PDGFRB
IGF1R
Cytokine-cytokine receptor interaction
Glial progenitor cell
PLCG1
IP_3
Ca^{2+}
CALM2
CAMK2B
PRKCB
DAG
+p
SHC4
GRB2
SOS2
KRAS
RAF1
MAP2K1
MAPK1
PIK3R1
PIP_3
AKT3
mTOR
PTEN
-p
ErbB signaling pathway
Calcium signaling pathway
MAPK signaling pathway
mTOR signaling pathway
Cell migration & mitosis
Cell survival
DNA
Cell growth & proliferation
MDM2
$p14^{ARF}$
p53
CDKN1A
CCND1
CDK6
RB1
E2F3
$p16^{INK4a}$
Cell cycle
G1/S progression
Genetic alterations
Oncogenes: EGFR, MDM2
Tumor suppressors: PTEN, IKN4a/ARF
Secondary pathway
Glial progenitor cell
Low-grade glioma
(WHO Grade II)
Anaplastic astrocytoma
(WHO Grade III)
Secondary GBM
(WHO Grade IV)
p53 signaling pathway
DNA damage
Impaired G1 and G2 arrest
Reduced apoptosis
Genomic instability
Genetic alterations
Oncogenes: PDGF, PDGFR, CDK4
Tumor suppressors: p53, Rb, PTEN

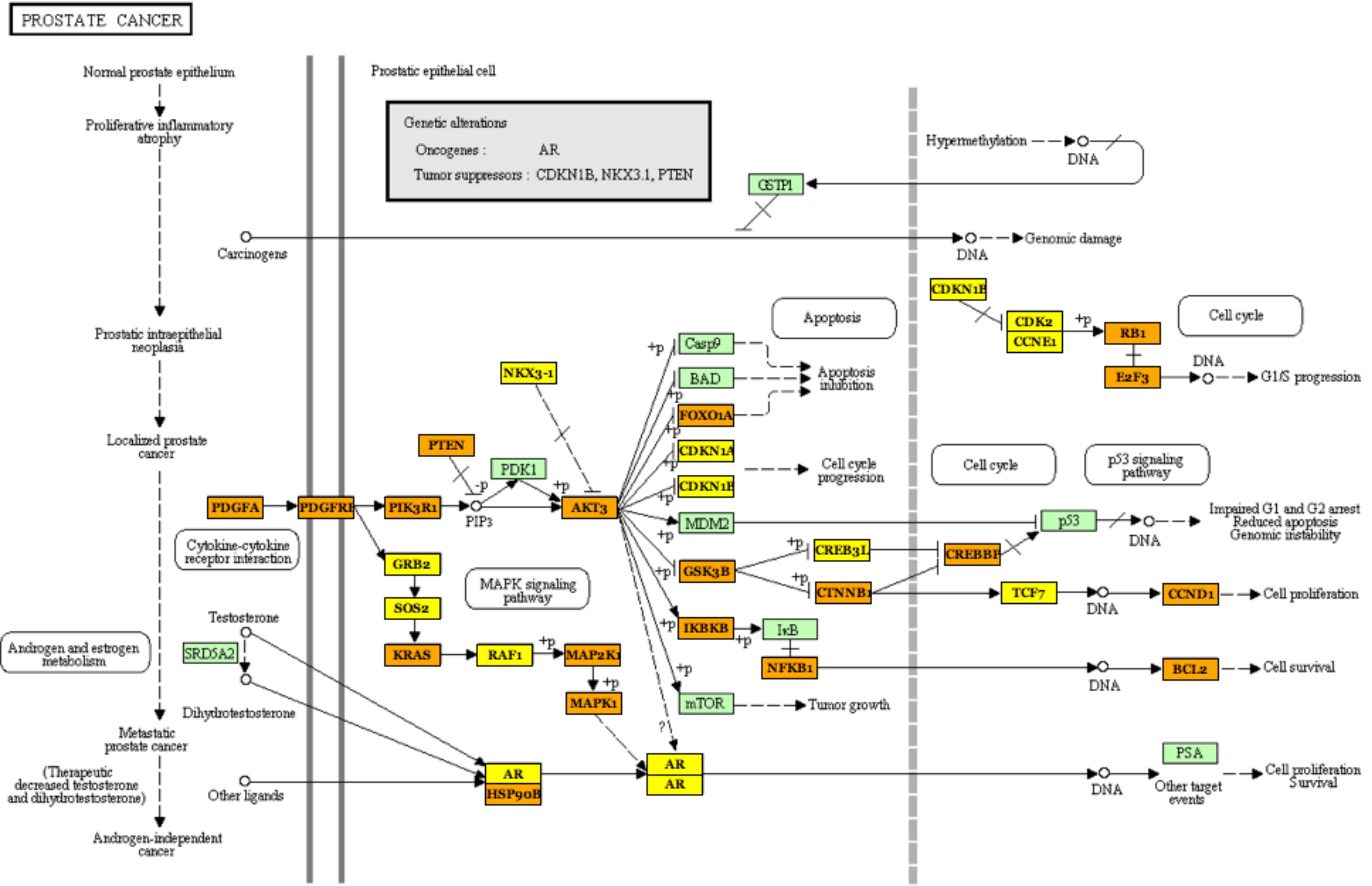
PROSTATE CANCER
Normal prostate epithelium
Proliferative inflammatory atrophy
Prostatic intraepithelial neoplasia
Localized prostate cancer
Metastatic prostate cancer
(Therapeutic decreased testosterone and dihydrotestosterone)
Androgen-independent cancer
Prostatic epithelial cell
Genetic alterations
Oncogenes : AR
Tumor suppressors : CDKN1B, NKX3.1, PTEN
Hypermethylation
DNA
GSTP1
Carcinogens
Genomic damage
CDKN1B
CDK2
CCNE1
RB1
E2F3
Cell cycle
G1/S progression
Apoptosis
Casp9
BAD
FOXO1A
CDKN1A
CDKN1B
Apoptosis inhibition
Cell cycle progression
NKX3-1
PTEN
PDK1
PDGFA
PDGFRB
PIK3R1
PIP3
AKT3
MDM2
p53
p53 signaling pathway
Impaired G1 and G2 arrest
Reduced apoptosis
Genomic instability
Cytokine-cytokine receptor interaction
GRB2
SOS2
KRAS
RAF1
MAP2K1
MAPK1
MAPK signaling pathway
CREB3L
CREBBP
GSK3B
CTNNB1
TCF7
CCND1
Cell proliferation
IKBKB
IκB
NFKB1
BCL2
Cell survival
mTOR
Tumor growth
Testosterone
SRD5A2
Dihydrotestosterone
Androgen and estrogen metabolism
Other ligands
AR
HSP90B
PSA
Other target events
Cell proliferation
Survival

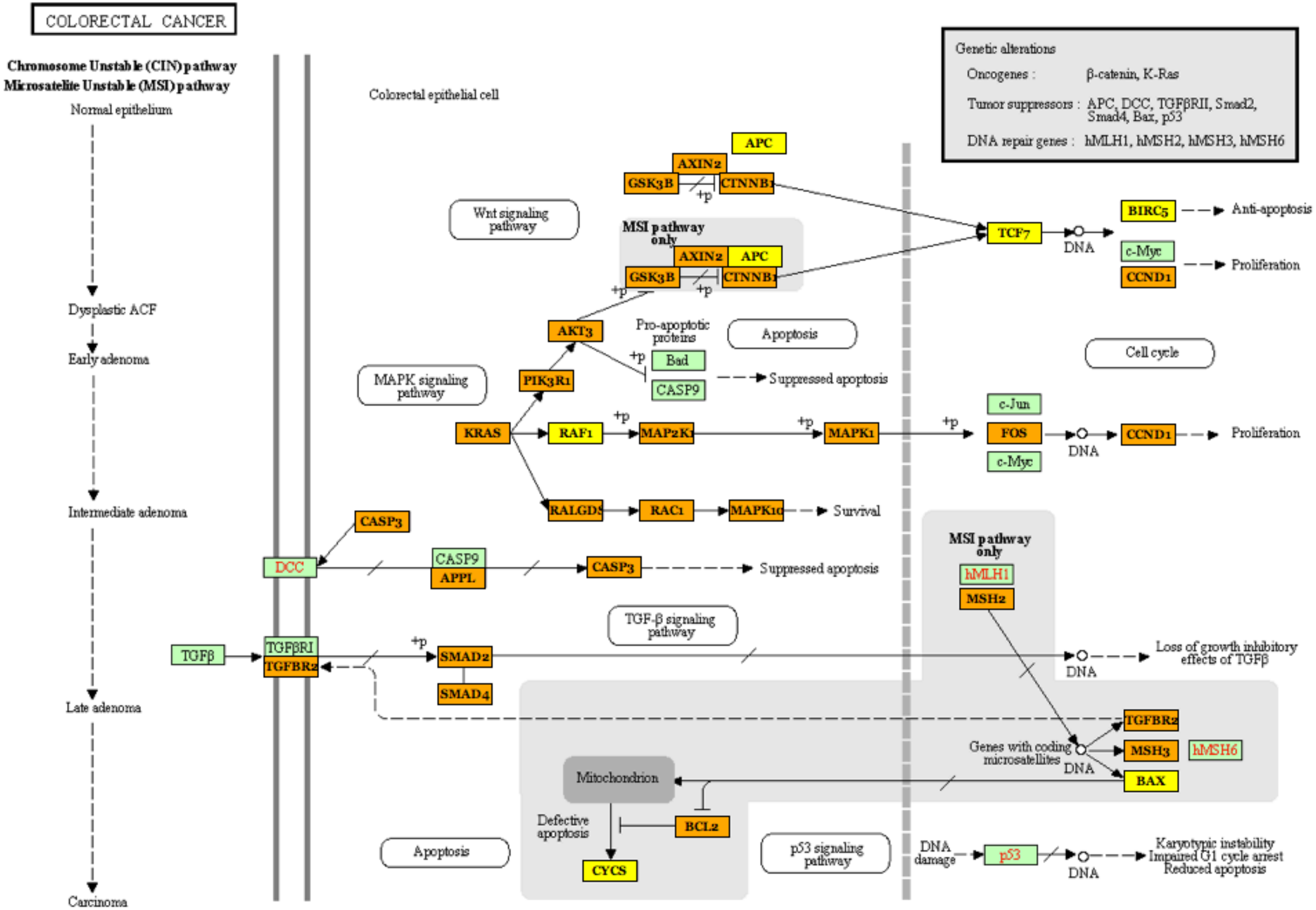

COLORECTAL CANCER
Chromosome Unstable (CIN) pathway
Microsatellite Unstable (MSI) pathway
Normal epithelium
Dysplastic ACF
Early adenoma
Intermediate adenoma
Late adenoma
Carcinoma
Colorectal epithelial cell
Genetic alterations
Oncogenes : β-catenin, K-Ras
Tumor suppressors : APC, DCC, TGFβRII, Smad2, Smad4, Bax, p53
DNA repair genes : hMLH1, hMSH2, hMSH3, hMSH6
APC
AXIN2
GSK3B
CTNNB1
+p
Wnt signaling pathway
MSI pathway only
AXIN2
APC
GSK3B
CTNNB1
TCF7
DNA
BIRC5
Anti-apoptosis
c-Myc
CCND1
Proliferation
AKT3
Pro-apoptotic proteins
Bad
CASP9
Suppressed apoptosis
Apoptosis
Cell cycle
MAPK signaling pathway
PIK3R1
KRAS
RAF1
MAP2K1
MAPK1
c-Jun
FOS
c-Myc
CCND1
Proliferation
RALGDS
RAC1
MAPK10
Survival
CASP3
DCC
CASP9
APPL
CASP3
Suppressed apoptosis
MSI pathway only
hMLH1
MSH2
TGF-β signaling pathway
TGFβ
TGFβRI
TGFBR2
SMAD2
SMAD4
Loss of growth inhibitory effects of TGFβ
TGFBR2
Genes with coding microsatellites
MSH3
hMSH6
BAX
Mitochondrion
Defective apoptosis
BCL2
CYCS
Apoptosis
p53 signaling pathway
DNA damage
p53
Karyotypic instability
Impaired G1 cycle arrest
Reduced apoptosis

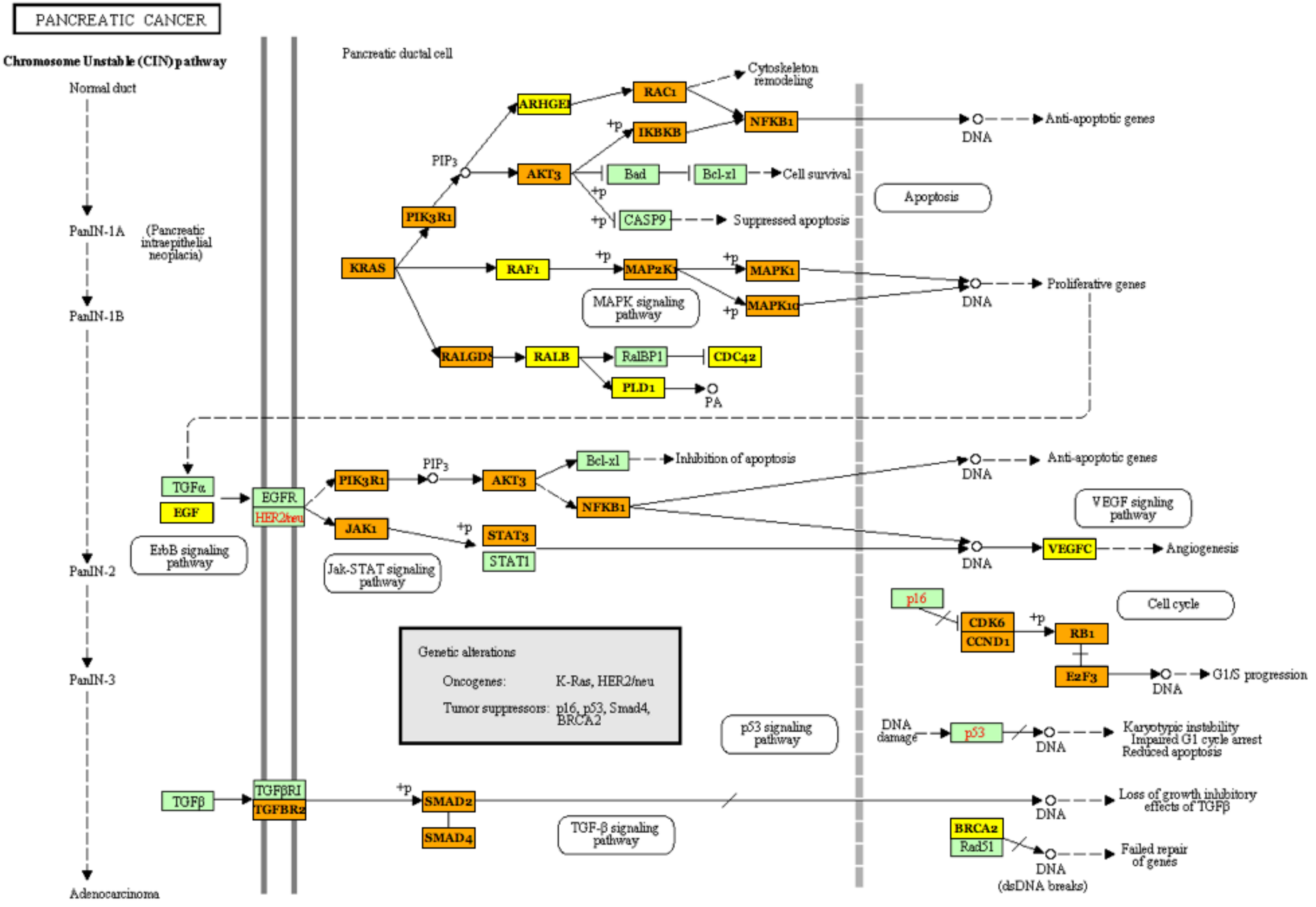
PANCREATIC CANCER
Chromosome Unstable (CIN) pathway
Normal duct
PanIN-1A
(Pancreatic intraepithelial neoplacia)
PanIN-1B
PanIN-2
PanIN-3
Adenocarcinoma
Pancreatic ductal cell
ARHGEF
RAC1
Cytoskeleton remodeling
NFKB1
IKBKB
DNA
Anti-apoptotic genes
PIP3
AKT3
Bad
Bcl-xl
Cell survival
PIK3R1
CASP9
Suppressed apoptosis
Apoptosis
KRAS
RAF1
MAP2K1
MAPK1
MAPK10
Proliferative genes
MAPK signaling pathway
RALGDS
RALB
RalBP1
CDC42
PLD1
PA
TGFα
EGF
EGFR
HER2/neu
ErbB signaling pathway
JAK1
STAT3
STAT1
Jak-STAT signaling pathway
Inhibition of apoptosis
VEGF signaling pathway
VEGFC
Angiogenesis
p16
CDK6
CCND1
RB1
E2F3
Cell cycle
G1/S progression
Genetic alterations
Oncogenes: K-Ras, HER2/neu
Tumor suppressors: p16, p53, Smad4, BRCA2
p53 signaling pathway
DNA damage
p53
Karyotypic instability
Impaired G1 cycle arrest
Reduced apoptosis
TGFβ
TGFβRI
TGFBR2
SMAD2
SMAD4
TGF-β signaling pathway
Loss of growth inhibitory effects of TGFβ
BRCA2
Rad51
Failed repair of genes
(dsDNA breaks)

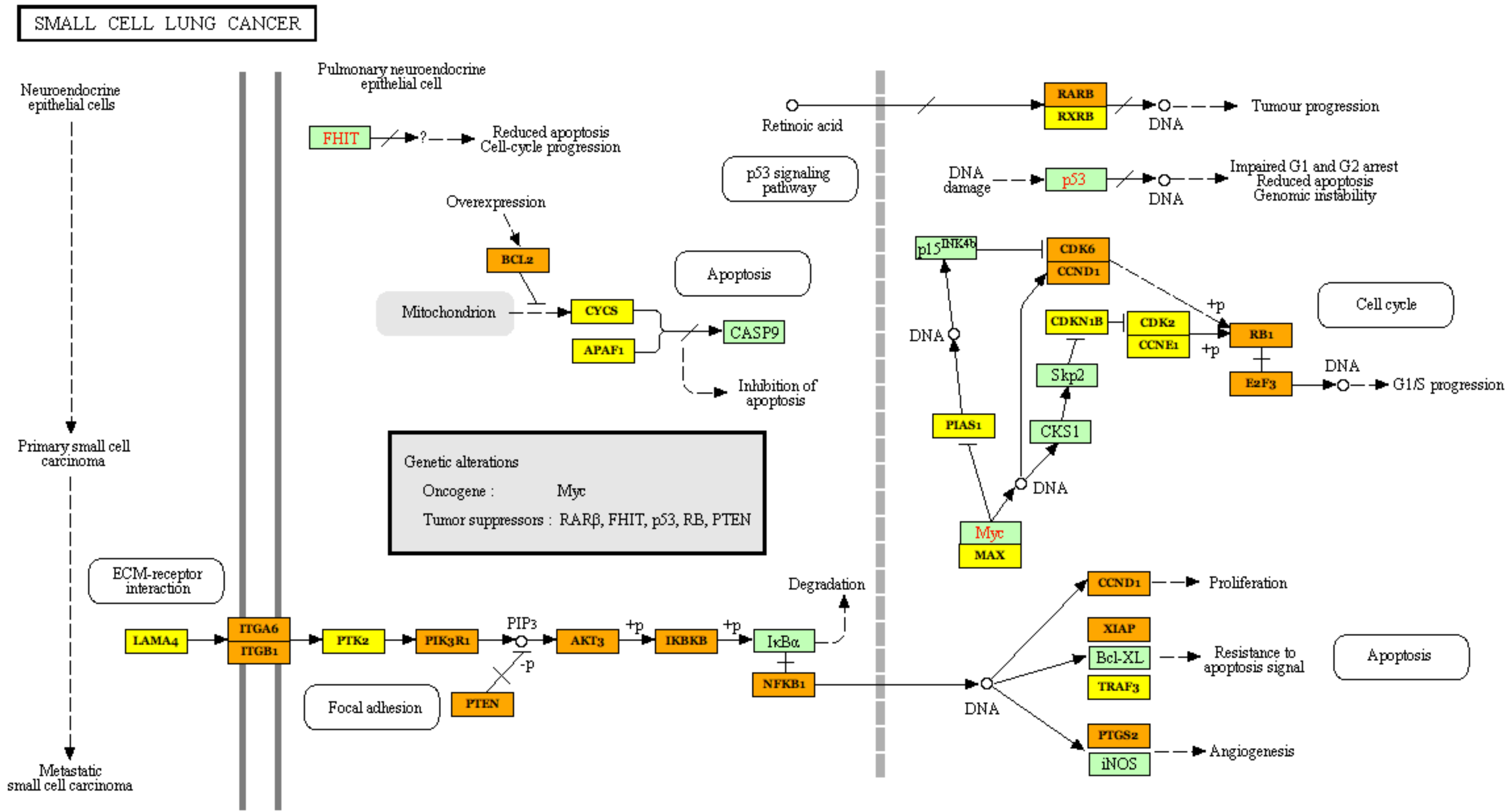
SMALL CELL LUNG CANCER
Neuroendocrine epithelial cells
Primary small cell carcinoma
Metastatic small cell carcinoma
Pulmonary neuroendocrine epithelial cell
FHIT
?
Reduced apoptosis
Cell-cycle progression
Overexpression
BCL2
Mitochondrion
CYCS
APAF1
CASP9
Apoptosis
Inhibition of apoptosis
p53 signaling pathway
Retinoic acid
RARB
RXRB
DNA
Tumour progression
DNA damage
p53
Impaired G1 and G2 arrest
Reduced apoptosis
Genomic instability
p15INK4b
CDK6
CCND1
CDKN1B
CDK2
CCNE1
+p
RB1
E2F3
G1/S progression
Cell cycle
Skp2
CKS1
PIAS1
Myc
MAX
Genetic alterations
Oncogene : Myc
Tumor suppressors : RARβ, FHIT, p53, RB, PTEN
ECM-receptor interaction
LAMA4
ITGA6
ITGB1
PTK2
PIK3R1
PIP3
-p
PTEN
Focal adhesion
AKT3
IKBKB
IκBα
Degradation
NFKB1
Proliferation
XIAP
Bcl-XL
TRAF3
Resistance to apoptosis signal
PTGS2
iNOS
Angiogenesis

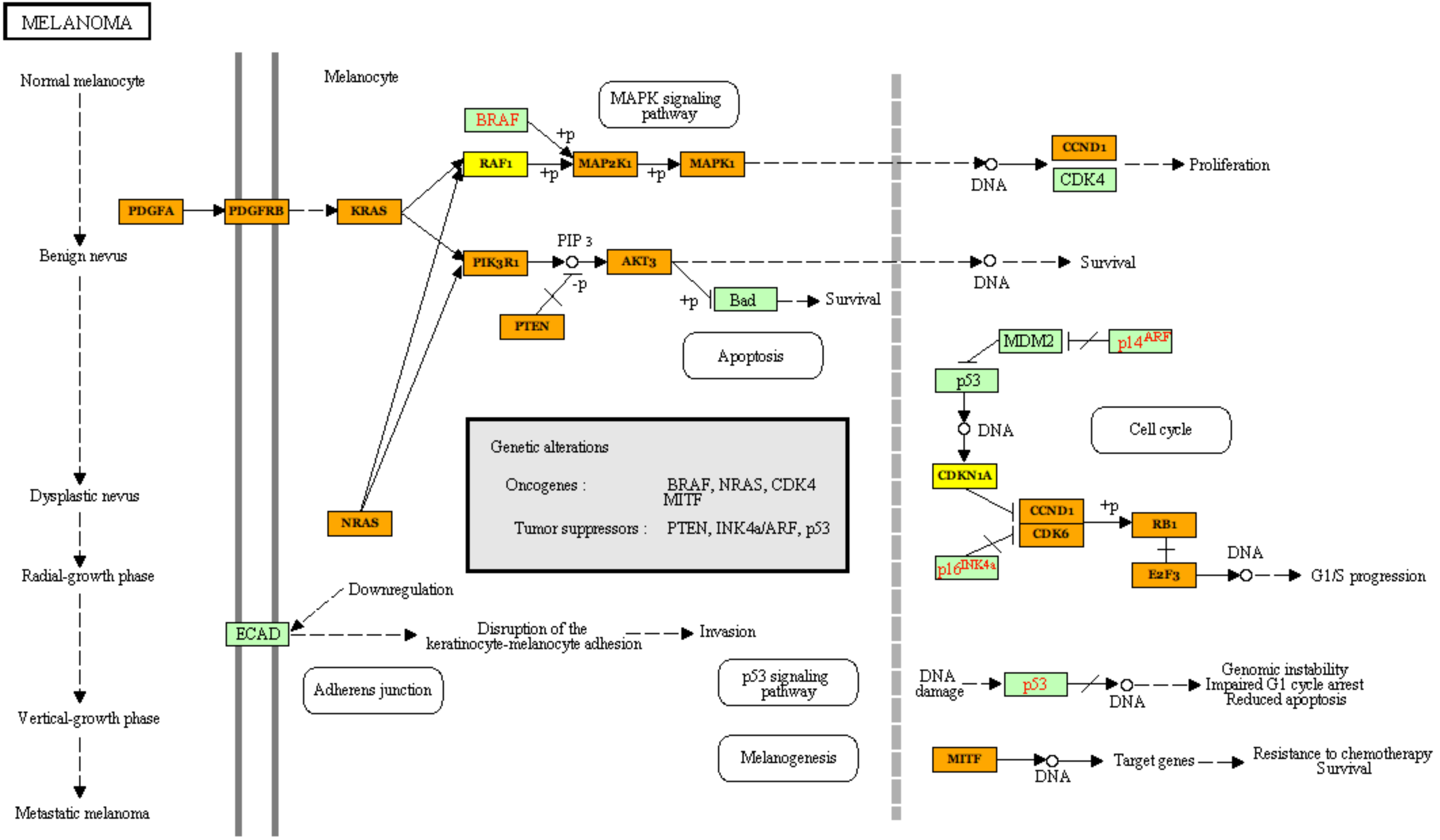
MELANOMA
Normal melanocyte
Benign nevus
Dysplastic nevus
Radial-growth phase
Vertical-growth phase
Metastatic melanoma
Melanocyte
PDGFA
PDGFRB
KRAS
NRAS
BRAF
RAF1
MAP2K1
MAPK1
+p
MAPK signaling pathway
PIK3R1
PIP 3
-p
PTEN
AKT3
Bad
Survival
Apoptosis
DNA
CCND1
CDK4
Proliferation
Survival
MDM2
p14ARF
p53
DNA
Cell cycle
CDKN1A
CCND1
CDK6
RB1
E2F3
p16INK4a
DNA
G1/S progression
Genetic alterations
Oncogenes : BRAF, NRAS, CDK4 MITF
Tumor suppressors : PTEN, INK4a/ARF, p53
Downregulation
ECAD
Disruption of the keratinocyte-melanocyte adhesion
Invasion
Adherens junction
p53 signaling pathway
Melanogenesis
DNA damage
p53
DNA
Genomic instability
Impaired G1 cycle arrest
Reduced apoptosis
MITF
DNA
Target genes
Resistance to chemotherapy
Survival
