# Supplementary material for: Non-Invasive microRNA Profiling in Saliva can Serve as a Biomarker of Alcohol Exposure and Its Effects in Humans
Source: Front Genet. 2022 Jan 20;12:804222. doi: 10.3389/fgene.2021.804222 (PMC8812725; doi:10.3389/fgene.2021.804222)
Supplement: Supplementary file 3 [file Image3.pdf]

NLK 3'UTR matches to miRNAs: miR-362, and -1825

```
TARGET  : NLK_3'UTR_ENST00000407008
length: 1745
MIRNA   : miR-362-3p
length: 22

mfe: -20.1 kcal/mol
p-value: undefined

position  1309
target 5'  U     AG   AAAACCUU       G 3'
            GGUCU  GAA        GGUGUGU
            UUAGG  CUU        CCACACA
miRNA  3' AC     AA   AU             A 5'
```

```
TARGET  : NLK_3'UTR_ENST00000407008
length: 1745
MIRNA   : miR-1825
length: 18

mfe: -21.4 kcal/mol
p-value: undefined

position  867
target 5' U    CUUUUCCU        C 3'
           GAGA        GCACUGGA
           CUCU        CGUGACCU
miRNA  3' C    CCUCC            5'
```

MET 3'UTR matches to miRNAs : miR-34a, -182, and -449a

```
TARGET  : MET_3'UTR_ENST00000318493
length: 218
MIRNA   : miR-34a-5p
length: 22

mfe: -21.9 kcal/mol
p-value: undefined

position  21
target 5' A    U  ACACUUUGUCCAAU    UUUU       U 3'
           ACAG CC              GGUU    CACUGCC
           UGUU GG              UCGA    GUGACGG
miRNA  3'                           UUCU       U 5'
```

TARGET : MET_3'UTR_ENST00000318493
length: 218
MIRNA : miR-182-5p
length: 24

mfe: -21.1 kcal/mol
p-value: undefined

position 83
```
target 5'         C      C        A 3'
                   UUUGCU UUGCCAAA
                   AGAUGG AACGGUUU
miRNA  3' UCACACUCA      U          5'
```

TARGET : MET_3'UTR_ENST00000318493
length: 218
MIRNA : miR-449a

length: 22

mfe: -20.7 kcal/mol
p-value: undefined

position 22
```
target 5'  A    CC   CUUUGUCCAAUG  UUUUU       U 3'
            CAGU  ACA            GU     CACUGCC
            GUCG  UGU            UA     GUGACGG
miRNA  3' UG    AU                 U           U 5'
```

TARGET : MET_3'UTR_ENST00000397752
length: 2262
MIRNA : miR-34a-5p
length: 22

mfe: -22.4 kcal/mol
p-value: undefined

position 2150
```
target 5'  G      AUACUU U       U 3'
            AAUUAG      G CACUGCC
            UUGGUC      C GUGACGG
miRNA  3' UG      GAUU   U       U 5'
```

TARGET : MET_3'UTR_ENST00000397752
length: 2262
MIRNA : miR-34a-5p
length: 22

mfe: -21.9 kcal/mol
p-value: undefined

position 21
```
target 5' A    U  ACACUUUGUCCAAU    UUUU       U 3'
           ACAG CC              GGUU    CACUGCC
           UGUU GG              UCGA    GUGACGG
miRNA  3'                           UUCU       U 5'
```

TARGET : MET_3'UTR_ENST00000397752
length: 2262
MIRNA : miR-34a-5p
length: 22

mfe: -19.3 kcal/mol
p-value: undefined

position 1842

```
target 5'  U  AG     UUACAAUCCAA         U 3'
            GA  AGCUA           AUAUUGCCG
            UU  UCGAU           UGUGACGGU
miRNA  3' UG  GG     UC                    5'
```

TARGET : MET_3'UTR_ENST00000397752
length: 2262
MIRNA : miR-182-5p
length: 24

mfe: -30.5 kcal/mol
p-value: undefined

position 684

```
target 5'     G           UG        G 3'
               GGGUUUUGCCA  UUGCCAAG
               CUCAAGAUGGU  AACGGUUU
miRNA  3' UCACA                      5'
```

TARGET : MET_3'UTR_ENST00000397752
length: 2262
MIRNA : miR-182-5p
length: 24

mfe: -21.6 kcal/mol
p-value: undefined

position 528

```
target 5'    U    ACAGGA   CACU           G 3'
              UGAG      UCU    CUGUUGCCAGG
              ACUC      AGA    GGUAACGGUUU
miRNA  3' UCAC    A        U                5'
```

TARGET : MET_3'UTR_ENST00000397752
length: 2262
MIRNA : miR-182-5p
length: 24

mfe: -21.5 kcal/mol
p-value: undefined

position 1446

```
target 5' U     A   UAC   A   GGUU       C 3'
           GGUGU GGU   UCU ACU    UUGUCGA
           UCACA UCA   AGA UGG    AACGGUU
miRNA  3'       C             U          U 5'
```

TARGET : MET_3'UTR_ENST00000397752
length: 2262
MIRNA : miR-182-5p
length: 24

mfe: -21.1 kcal/mol
p-value: undefined

position 83

```
target 5'         C      C        A 3'
                   UUUGCU UUGCCAAA
                   AGAUGG AACGGUUU
miRNA  3' UCACACUCA      U          5'
```

TARGET : MET_3'UTR_ENST00000397752
length: 2262
MIRNA : miR-182-5p
length: 24

mfe: -19.6 kcal/mol
p-value: undefined

position 1642

```
target 5'      G  AA G    G        U 3'
               GA  U UACU AUUGCCAA
               CU  A AUGG UAACGGUU
miRNA  3' UCACA  CA G              U 5'
```

TARGET : MET_3'UTR_ENST00000397752
length: 2262
MIRNA : miR-449a
length: 22

mfe: -20.7 kcal/mol
p-value: undefined

position 22

```
target 5'  A    CC   CUUUGUCCAAUG  UUUUU       U 3'
            CAGU  ACA            GU     CACUGCC
            GUCG  UGU            UA     GUGACGG
miRNA  3' UG    AU                 U           U 5'
```

TARGET : MET_3'UTR_ENST00000397752
length: 2262
MIRNA : miR-449a
length: 22

mfe: -22.0 kcal/mol
p-value: undefined

position 2151

```
target 5' A     AU   UUGU       U 3'
           AUUAG   AC    CACUGCC
           UGGUC   UG    GUGACGG
miRNA  3'       GAU  UUAU       U 5'
```

TARGET : MET_3’UTR_ENST00000397752
length: 2262
MIRNA : miR-449a
length: 22

mfe: -25.1 kcal/mol
p-value: undefined

position 1846

```
target 5'   G     UU     CCAA         U 3'
             AGCUA  ACAAU    AUAUUGCCG
             UCGAU  UGUUA    UGUGACGGU
miRNA  3' UGG                          5'
```

SNAi1 3’UTR matches to miRNAs : miR-30b, 34a, and 449a

TARGET : SNAi1_3'UTR_ENST00000244050
length: 830
MIRNA : miR-30b-5p
length: 22

mfe: -24.1 kcal/mol
p-value: undefined

position 685

```
target 5' C  G    CC  GG    AGA        U 3'
           AG UGGG  UG  AGGA   UGUUUACA
           UC ACUC  AC  UCCU   ACAAAUGU
miRNA  3'    G        A                 5'
```

TARGET : SNAi1_3'UTR_ENST00000244050
length: 830
MIRNA : miR-34a-5p
length: 22

mfe: -27.7 kcal/mol
p-value: undefined

position 55

```
target 5' A  U      AGG    ACCCCACAUCCUUCU        U 3'
           GC  CCAGC   AAGG               CACUGCCA
           UG  GGUCG   UUCU               GUGACGGU
miRNA  3'    UU     A                              5'
```

TARGET : SNAi1_3'UTR_ENST00000244050
length: 830
MIRNA : miR-449a
length: 22

mfe: -27.6 kcal/mol
p-value: undefined

position 52

```
target 5' C      CC     GAAG ACCCCACAUCCUUCU        U 3'
           CCAGCU  AGCAG    G                CACUGCCA
           GGUCGA  UUGUU    U                GUGACGGU
miRNA  3' U            A                             5'
```

CTNND1 3’UTR matches to miRNAs : miR-34a, -449a, and -548b

TARGET : CTNND1_3'UTR_ENST00000428599
length: 2870
MIRNA : miR-34a-5p
length: 22

mfe: -25.9 kcal/mol
p-value: undefined

position 684

```
target 5' C     CUG     UU C       C 3'
           CAACC   GGCUA  G UGCUGCC
           GUUGG   UCGAU  C GUGACGG
miRNA  3' U             U  U       U 5'
```

TARGET : CTNND1_3'UTR_ENST00000428599
length: 2870
MIRNA : miR-34a-5p
length: 22

mfe: -20.4 kcal/mol
p-value: undefined

position 2353

```
target 5'  A UG      AAGUUGAACAUG   GCU        A 3'
            A  UGGCUG            GGA   UAUUGCUA
            U  GUCGAU            UCU   GUGACGGU
miRNA  3' UG UG                                5'
```

TARGET : CTNND1_3'UTR_ENST00000428599
length: 2870
MIRNA : miR-449a
length: 22

mfe: -21.6 kcal/mol
p-value: undefined

position 686

```
target 5' A   CUG     UUGC         C 3'
           ACC   GGCUA      UGCUGCC
           UGG   UCGAU      GUGACGG
miRNA  3'             UGUUAU       U 5'
```

TARGET : CTNND1_3'UTR_ENST00000428599
length: 2870
MIRNA : miR-548b-5p
length: 22

mfe: -19.1 kcal/mol
p-value: undefined

position 23

```
target 5' G    UAU UG   UU        U 3'
           GGCU   A  UAC  UUAUUUUU
           CCGG   U  GUG  AAUGAAAA
miRNA  3'      UU  UG   UU          5'
```

References:

Marc Rehmsmeier *, Peter Steffen, Matthias Höchsmann, Robert Giegerich (2004) Fast and effective prediction of microRNA/target duplexes. *RNA*, 10:1507-1517.

Vlachos IS, Kostoulas N, Vergoulis T, Georgakilas G, Reczko M, Maragkakis M, Paraskevopoulou MD, Prionidis K, Dalamagas T, Hatzigeorgiou AG (2012) DIANA miRPath v.2.0: investigating the combinatorial effect of microRNAs in pathways. *Nucleic Acids Res.*, W498-W504.
