# Supplementary material for: Non-Invasive microRNA Profiling in Saliva can Serve as a Biomarker of Alcohol Exposure and Its Effects in Humans
Source: Front Genet. 2022 Jan 20;12:804222. doi: 10.3389/fgene.2021.804222 (PMC8812725; doi:10.3389/fgene.2021.804222)
Supplement: Supplementary file 4 [file Table2.docx]

**Supplementary Table S2. Correlation of the array and qPCR results.**

Ten random microRNAs (representing each microRNA change group: downregulation [4 microRNAs], upregulation [3 microRNAs], expression de novo [3 microRNAs]) per each sample group (3 controls, 3 EtOH) have been selected. Each result is an average CT of duplicate (array) or triplicate (qPCR) measurements before normalization to U6. qPCR CT and array CT show strong, positive correlation, r(58) = .89, *p* < 0.0001

| # | **sample** | **microRNA change** | **Sample #** | **microRNA** | **qPCR CT** | **array CT** |
| --- | --- | --- | --- | --- | --- | --- |
| 1 | Ctrl | downregulation | Sample 1 | miR-106a | 18.77 | 20.95 |
| 2 | Ctrl | downregulation | Sample 2 | miR-106a | 22.66 | 22.66 |
| 3 | Ctrl | downregulation | Sample 3 | miR-106a | 22.66 | 22.68 |
| 4 | Ctrl | downregulation | Sample 1 | miR-17 | 19.01 | 20.96 |
| 5 | Ctrl | downregulation | Sample 2 | miR-17 | 22.76 | 22.54 |
| 6 | Ctrl | downregulation | Sample 3 | miR-17 | 22.74 | 22.53 |
| 7 | Ctrl | downregulation | Sample 1 | miR-29a | 20.89 | 22.94 |
| 8 | Ctrl | downregulation | Sample 2 | miR-29a | 23.65 | 22.9 |
| 9 | Ctrl | downregulation | Sample 3 | miR-29a | 24.51 | 23.62 |
| 10 | Ctrl | downregulation | Sample 2 | miR-618 | 28.3 | 27 |
| 11 | Ctrl | downregulation | Sample 5 | miR-618 | 29.2 | 27.9 |
| 12 | Ctrl | downregulation | Sample 6 | miR-618 | 29.2 | 27.2 |
| 13 | Ctrl | expression de novo | Sample 1 | miR-1 | 40 | 40 |
| 14 | Ctrl | expression de novo | Sample 2 | miR-1 | 40 | 40 |
| 15 | Ctrl | expression de novo | Sample 3 | miR-1 | 31.98 | 40 |
| 16 | Ctrl | expression de novo | Sample 7 | miR-10a | 40 | 40 |
| 17 | Ctrl | expression de novo | Sample 10 | miR-10a | 40 | 40 |
| 18 | Ctrl | expression de novo | Sample 16 | miR-10a | 29.74 | 40 |
| 19 | Ctrl | expression de novo | Sample 7 | miR-182 | 33.7 | 40 |
| 20 | Ctrl | expression de novo | Sample 10 | miR-182 | 35.83 | 40 |
| 21 | Ctrl | expression de novo | Sample 16 | miR-182 | 33.62 | 40 |
| 22 | Ctrl | upregulation | Sample 1 | miR-20a | 19.5 | 20.96 |
| 23 | Ctrl | upregulation | Sample 2 | miR-20a | 22.23 | 22.09 |
| 24 | Ctrl | upregulation | Sample 3 | miR-20a | 22.47 | 22.21 |
| 25 | Ctrl | upregulation | Sample 1 | miR-26a | 18.8 | 20.96 |
| 26 | Ctrl | upregulation | Sample 2 | miR-26a | 21.68 | 22.79 |
| 27 | Ctrl | upregulation | Sample 3 | miR-26a | 22.91 | 23.98 |
| 28 | Ctrl | upregulation | Sample 1 | miR-27a | 20.54 | 22.97 |
| 29 | Ctrl | upregulation | Sample 2 | miR-27a | 22.81 | 22.42 |
| 30 | Ctrl | upregulation | Sample 3 | miR-27a | 22.9 | 22.8 |
| 31 | EtOH | downregulation | Sample 7 | miR-106a | 25.85 | 24.37 |
| 32 | EtOH | downregulation | Sample 10 | miR-106a | 24.37 | 24.03 |
| 33 | EtOH | downregulation | Sample 16 | miR-106a | 21.83 | 22.01 |
| 34 | EtOH | downregulation | Sample 7 | miR-17 | 25.92 | 24.27 |
| 35 | EtOH | downregulation | Sample 10 | miR-17 | 24.59 | 23.94 |
| 36 | EtOH | downregulation | Sample 16 | miR-17 | 22.05 | 21.98 |
| 37 | EtOH | downregulation | Sample 7 | miR-29a | 26.83 | 24.39 |
| 38 | EtOH | downregulation | Sample 10 | miR-29a | 26.91 | 25.42 |
| 39 | EtOH | downregulation | Sample 16 | miR-29a | 22.57 | 21.92 |
| 40 | EtOH | downregulation | Sample 2 | miR-618 | 34.1 | 39.2 |
| 41 | EtOH | downregulation | Sample 7 | miR-618 | 34.9 | 33.6 |
| 42 | EtOH | downregulation | Sample 18 | miR-618 | 31.5 | 29.6 |
| 43 | EtOH | expression de novo | Sample 7 | miR-1 | 35.73 | 40 |
| 44 | EtOH | expression de novo | Sample 10 | miR-1 | 40 | 32.68 |
| 45 | EtOH | expression de novo | Sample 16 | miR-1 | 37.75 | 36.27 |
| 46 | EtOH | expression de novo | Sample 1 | miR-10a | 26.81 | 29.99 |
| 47 | EtOH | expression de novo | Sample 2 | miR-10a | 31.97 | 40 |
| 48 | EtOH | expression de novo | Sample 3 | miR-10a | 29.53 | 36.17 |
| 49 | EtOH | expression de novo | Sample 1 | miR-182 | 25.46 | 30 |
| 50 | EtOH | expression de novo | Sample 2 | miR-182 | 33.72 | 35.58 |
| 51 | EtOH | expression de novo | Sample 3 | miR-182 | 31.77 | 35.66 |
| 52 | EtOH | upregulation | Sample 7 | miR-20a | 27.95 | 26.18 |
| 53 | EtOH | upregulation | Sample 10 | miR-20a | 25.86 | 26.41 |
| 54 | EtOH | upregulation | Sample 16 | miR-20a | 21.9 | 21.78 |
| 55 | EtOH | upregulation | Sample 7 | miR-26a | 26.01 | 25.52 |
| 56 | EtOH | upregulation | Sample 10 | miR-26a | 25.74 | 26.84 |
| 57 | EtOH | upregulation | Sample 16 | miR-26a | 20.48 | 21.56 |
| 58 | EtOH | upregulation | Sample 7 | miR-27a | 25.57 | 23.41 |
| 59 | EtOH | upregulation | Sample 10 | miR-27a | 24.33 | 22.51 |
| 60 | EtOH | upregulation | Sample 16 | miR-27a | 22.96 | 22.61 |
