# Supplementary material for: Non-Invasive microRNA Profiling in Saliva can Serve as a Biomarker of Alcohol Exposure and Its Effects in Humans
Source: Front Genet. 2022 Jan 20;12:804222. doi: 10.3389/fgene.2021.804222 (PMC8812725; doi:10.3389/fgene.2021.804222)
Supplement: Supplementary file 5 [file Table3.docx]

**Supplementary Figure Legends**

**Supplementary Figure S1**

**Wiring diagrams of cancers from Table 3**: glioma (KEGG: hsa05214), prostate cancer (KEGG: hsa05215), colorectal cancer (KEGG: hsa05210), pancreatic cancer (KEGG: hsa05212), small cell lung carcinoma (KEGG: hsa05222) and melanoma (KEGG: hsa05218). Gene products in yellow boxes are affected by one microRNA, gene products in orange boxes are affected by more then one microRNA. Gene products in green boxes are unaffected. The wiring diagrams were created using KEGG (Kyoto Encyclopedia of Genes and Genomes) and DIANA-mirPath software with FDR correction and P value threshold of 0.05.

**Supplementary Figure S2**

**RNAhybrid binding prediction of microRNAs to selected adherens junction elements.** Four targets (NLK, MET, SNAi1, and CTNND1) were selected for expression analysis based upon their involvement in the Adherens Junction pathway. All four had been predicted by DIANA (Vlachos et al, 2012) to have binding sites for miRNAs that we had observed to increase in expression in alcohol abusers. RNAhybrid (Rehmsmeier et al., 2004) was used to examine more closely binding properties of these microRNA to sites in the 3’UTRs (obtained from Ensembl) of these transcripts. Settings adjusted include hits per target (10), energy cutoff (-19), and helix constraint (2-8), to display only hits with a strong likelihood of being valid binding sites.

**Supplementary Figure S3**

**Remaining wiring diagrams of cellular pathways described in Table 2**: endocytosis (KEGG: hsa04144), Wnt signaling (KEGG: hsa04310), MAPK signaling (KEGG: hsa04010) and ErbB signaling (KEGG: hsa04012). Gene products in yellow boxes are affected by one microRNA, gene products in orange boxes are affected by more than one microRNA. Gene products in green boxes are unaffected. The wiring diagrams were created using KEGG (Kyoto Encyclopedia of Genes and Genomes) and DIANA-mirPath software with FDR correction and P value threshold of 0.05.
