# Supplementary material for: Non-Invasive microRNA Profiling in Saliva can Serve as a Biomarker of Alcohol Exposure and Its Effects in Humans
Source: Front Genet. 2022 Jan 20;12:804222. doi: 10.3389/fgene.2021.804222 (PMC8812725; doi:10.3389/fgene.2021.804222)
Supplement: Supplementary file 6 [file Image1.pdf]

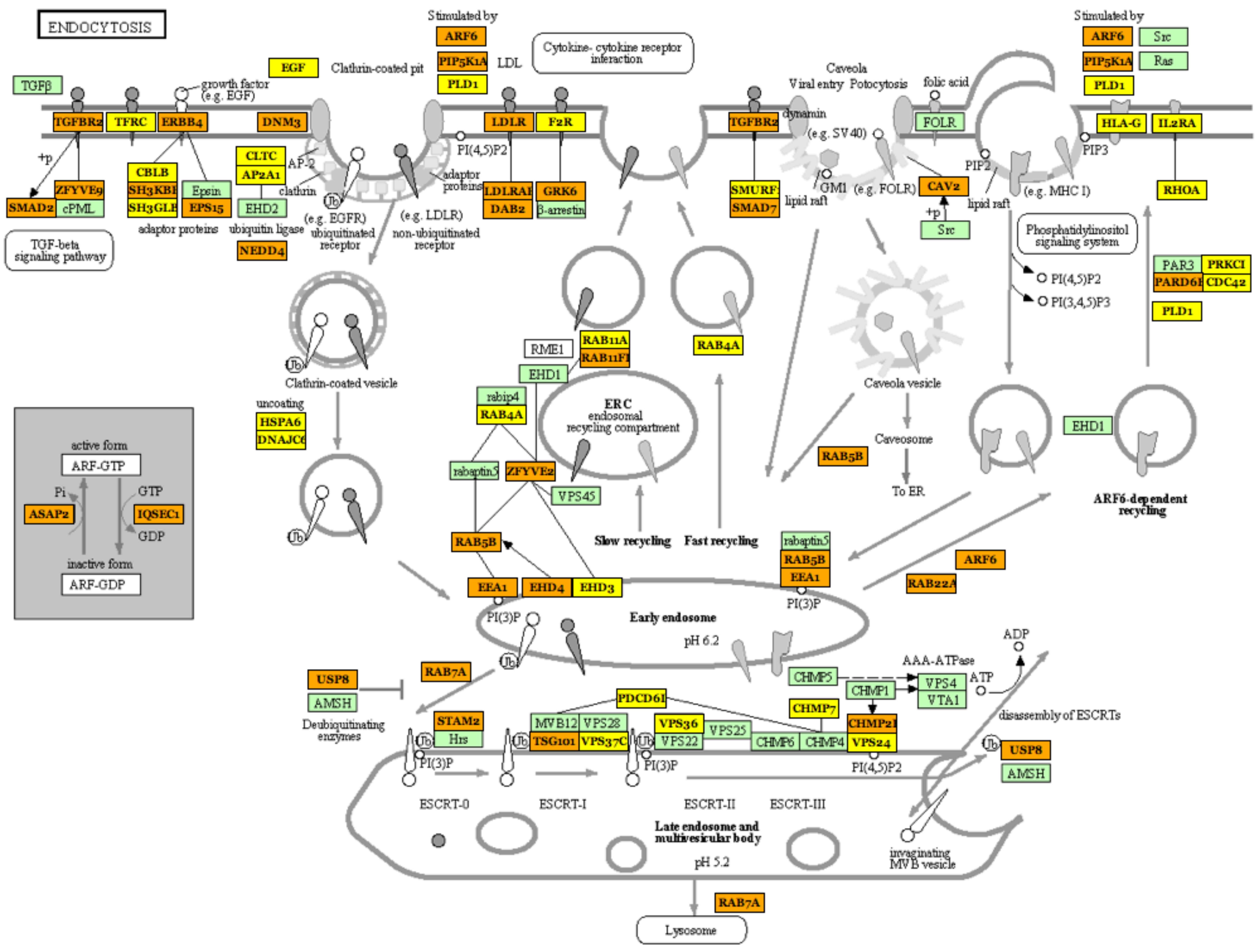

ENDOCYTOSIS
Stimulated by
ARF6
PIP5K1A
PLD1
LDL
EGF
Clathrin-coated pit
Cytokine- cytokine receptor interaction
TGFβ
growth factor (e.g. EGF)
TGFBR2
TFRC
ERBB4
DNM3
LDLR
F2R
+p
ZFYVE9
SMAD2
cPML
CBLB
SH3KBI
SH3GLB
Epsin
EPS15
CLTC
AP2A1
AP-2
clathrin
EHD2
NEDD4
adaptor proteins
ubiquitin ligase
(e.g. EGFR) ubiquitinated receptor
(e.g. LDLR) non-ubiquitinated receptor
adaptor proteins
PI(4,5)P2
LDLRAP
DAB2
GRK6
β-arrestin
TGF-beta signaling pathway
Ub
Clathrin-coated vesicle
uncoating
HSPA6
DNAJC6
active form
ARF-GTP
Pi
GTP
ASAP2
IQSEC1
GDP
inactive form
ARF-GDP
Caveola
Viral entry Potocytosis
folic acid
dynamin
(e.g. SV40)
FOLR
SMURF1
SMAD7
GM1
lipid raft
(e.g. FOLR)
CAV2
Src
PIP2
lipid raft
(e.g. MHC I)
Stimulated by
Src
Ras
HLA-G
IL2RA
PIP3
RHOA
Phosphatidylinositol signaling system
PI(4,5)P2
PI(3,4,5)P3
PAR3
PRKCI
PARD6B
CDC42
PLD1
RME1
RAB11A
RAB11FI
RAB4A
EHD1
rabip4
RAB4A
ERC endosomal recycling compartment
rabaptin5
ZFYVE2
VPS45
RAB5B
Slow recycling
Fast recycling
Caveola vesicle
Caveosome
To ER
RAB5B
EHD1
ARF6-dependent recycling
rabaptin5
RAB5B
EEA1
PI(3)P
ARF6
RAB22A
EEA1
EHD4
EHD3
PI(3)P
Early endosome
pH 6.2
USP8
AMSH
Deubiquitinating enzymes
RAB7A
STAM2
Hrs
MVB12
VPS28
TSG101
VPS37C
PDCD6I
VPS36
VPS22
VPS25
CHMP6
CHMP4
CHMP7
CHMP5
CHMP1
CHMP2
VPS24
VPS4
VTA1
AAA-ATPase
ATP
ADP
disassembly of ESCRTs
USP8
AMSH
PI(3)P
PI(3)P
PI(4,5)P2
ESCRT-0
ESCRT-I
ESCRT-II
ESCRT-III
Late endosome and multivesicular body
pH 5.2
invaginating MVB vesicle
RAB7A
Lysosome

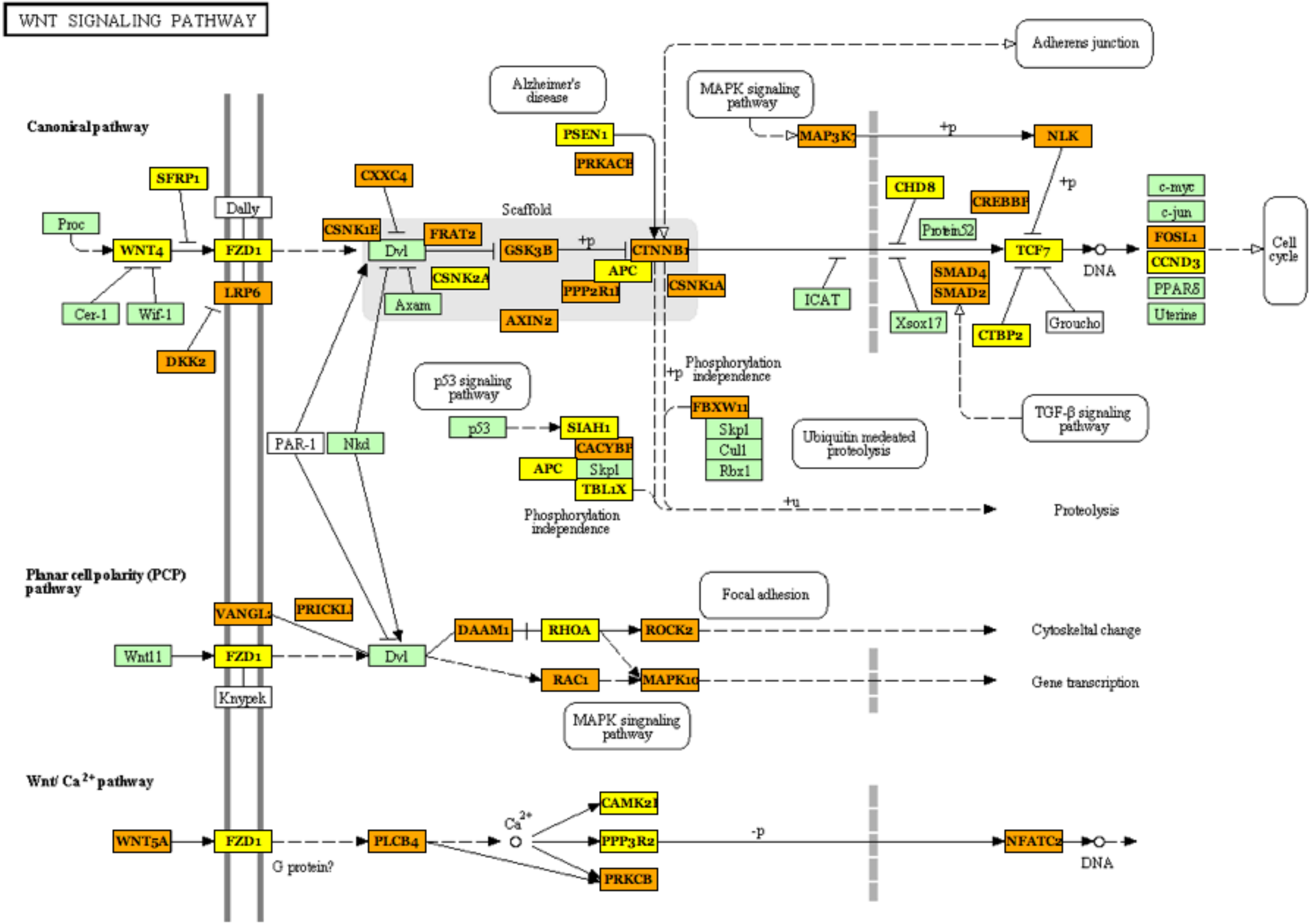
WNT SIGNALING PATHWAY
Canonical pathway
Proc
WNT4
SFRP1
Dally
FZD1
LRP6
Cer-1
Wif-1
DKK2
CXXC4
CSNK1E
FRAT2
Dvl
Axam
CSNK2A
Scaffold
GSK3B
+p
CTNNB1
APC
PPP2R1
AXIN2
CSNK1A
Alzheimer's disease
PSEN1
PRKACB
MAPK signaling pathway
MAP3K7
+p
NLK
+p
Adherens junction
CHD8
CREBBP
Protein52
ICAT
Xsox17
SMAD4
SMAD2
TCF7
CTBP2
Groucho
DNA
c-myc
c-jun
FOSL1
CCND3
PPARδ
Uterine
Cell cycle
p53 signaling pathway
p53
SIAH1
CACYBP
APC
Skp1
TBL1X
Phosphorylation independence
+p
Phosphorylation independence
FBXW11
Skp1
Cul1
Rbx1
Ubiquitin mediated proteolysis
TGF-β signaling pathway
+u
Proteolysis
PAR-1
Nkd
Planar cell polarity (PCP) pathway
VANGL2
PRICKLE
Wnt11
FZD1
Knypek
Dvl
DAAM1
RHOA
ROCK2
Focal adhesion
Cytoskeltal change
RAC1
MAPK10
Gene transcription
MAPK singnaling pathway
Wnt/ Ca2+ pathway
WNT5A
FZD1
G protein?
PLCB4
Ca2+
CAMK2B
PPP3R2
PRKCB
-p
NFATC2
DNA

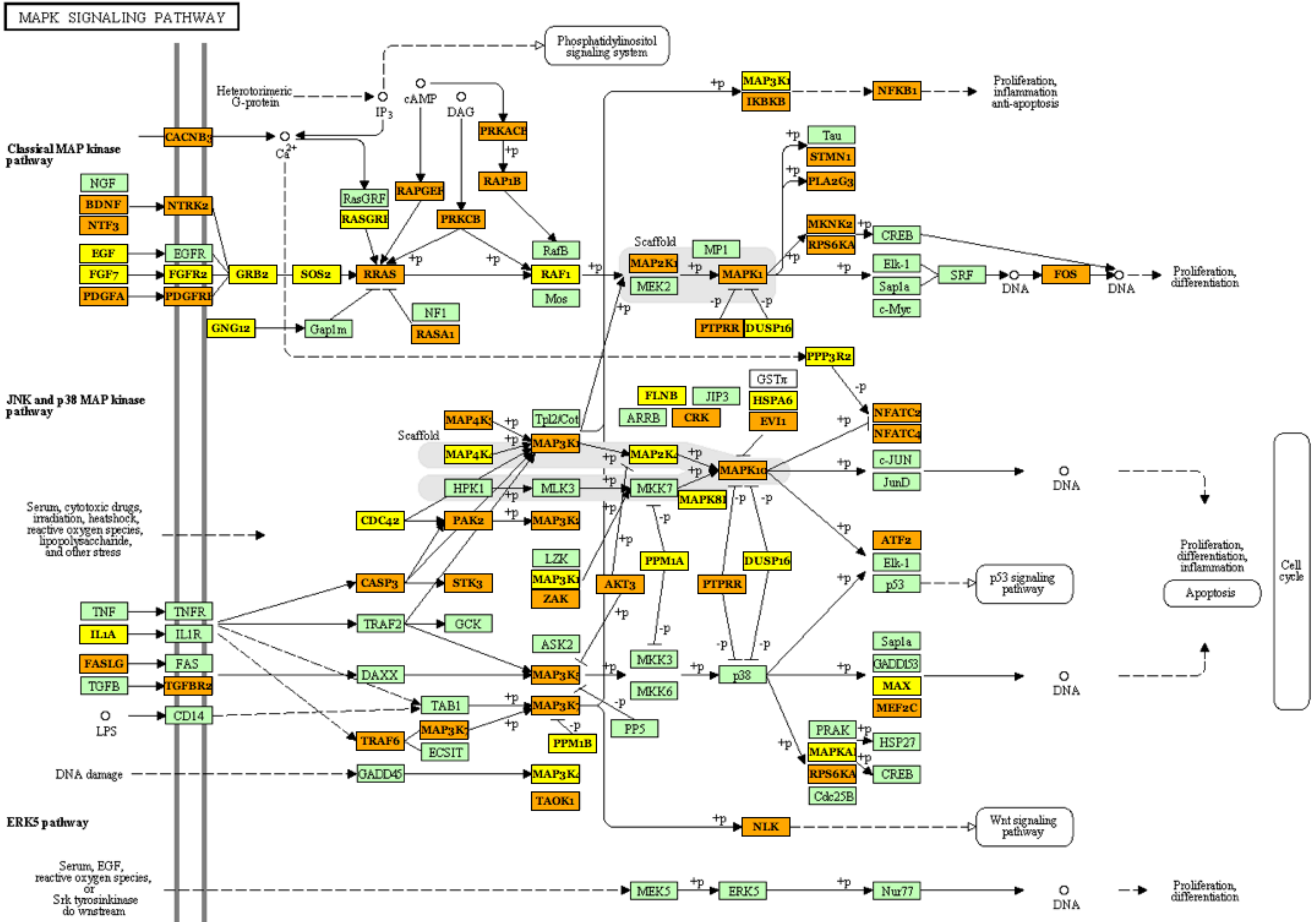
MAPK SIGNALING PATHWAY
Phosphatidylinositol signaling system
Heterotorimeric G-protein
IP3
cAMP
DAG
CACNB3
Ca2+
PRKACB
+p
RAP1B
Classical MAP kinase pathway
NGF
BDNF
NTF3
NTRK2
RasGRF
RASGRF
RAPGEF
PRKCB
RafB
EGF
EGFR
FGF7
FGFR2
PDGFA
PDGFRB
GRB2
SOS2
RRAS
RAF1
Mos
NF1
RASA1
GNG12
Gap1m
Scaffold
MP1
MAP2K1
MEK2
MAPK1
-p
PTPRR
DUSP16
MAP3K1
IKBKB
NFKB1
Proliferation, inflammation anti-apoptosis
Tau
STMN1
PLA2G3
MKNK2
RPS6KA
CREB
Elk-1
Sap1a
c-Myc
SRF
DNA
FOS
Proliferation, differentiation
PPP3R2
JNK and p38 MAP kinase pathway
FLNB
JIP3
GSTπ
HSPA6
ARRB
CRK
EVI1
NFATC2
NFATC4
MAP4K
MAP4K4
Tpl2/Cot
MAP3K1
MAP2K4
MAPK10
c-JUN
JunD
HPK1
MLK3
MKK7
MAPK8I
CDC42
PAK2
MAP3K2
Serum, cytotoxic drugs, irradiation, heatshock, reactive oxygen species, lipopolysaccharide, and other stress
LZK
MAP3K1
ZAK
PPM1A
DUSP16
AKT3
PTPRR
ATF2
Elk-1
p53
p53 signaling pathway
Proliferation, differentiation, inflammation
Apoptosis
Cell cycle
CASP3
STK3
TNF
TNFR
IL1A
IL1R
TRAF2
GCK
ASK2
FASLG
FAS
DAXX
MAP3K5
MKK3
p38
Sap1a
GADD153
MAX
MEF2C
TGFB
TGFBR2
MKK6
TAB1
MAP3K7
PP5
LPS
CD14
TRAF6
MAP3K7
ECSIT
PPM1B
PRAK
HSP27
MAPKAP
RPS6KA
CREB
Cdc25B
DNA damage
GADD45
MAP3K4
TAOK1
ERK5 pathway
NLK
Wnt signaling pathway
Serum, EGF, reactive oxygen species, or Srk tyrosinkinase do wnstream
MEK5
ERK5
Nur77
Proliferation, differentiation

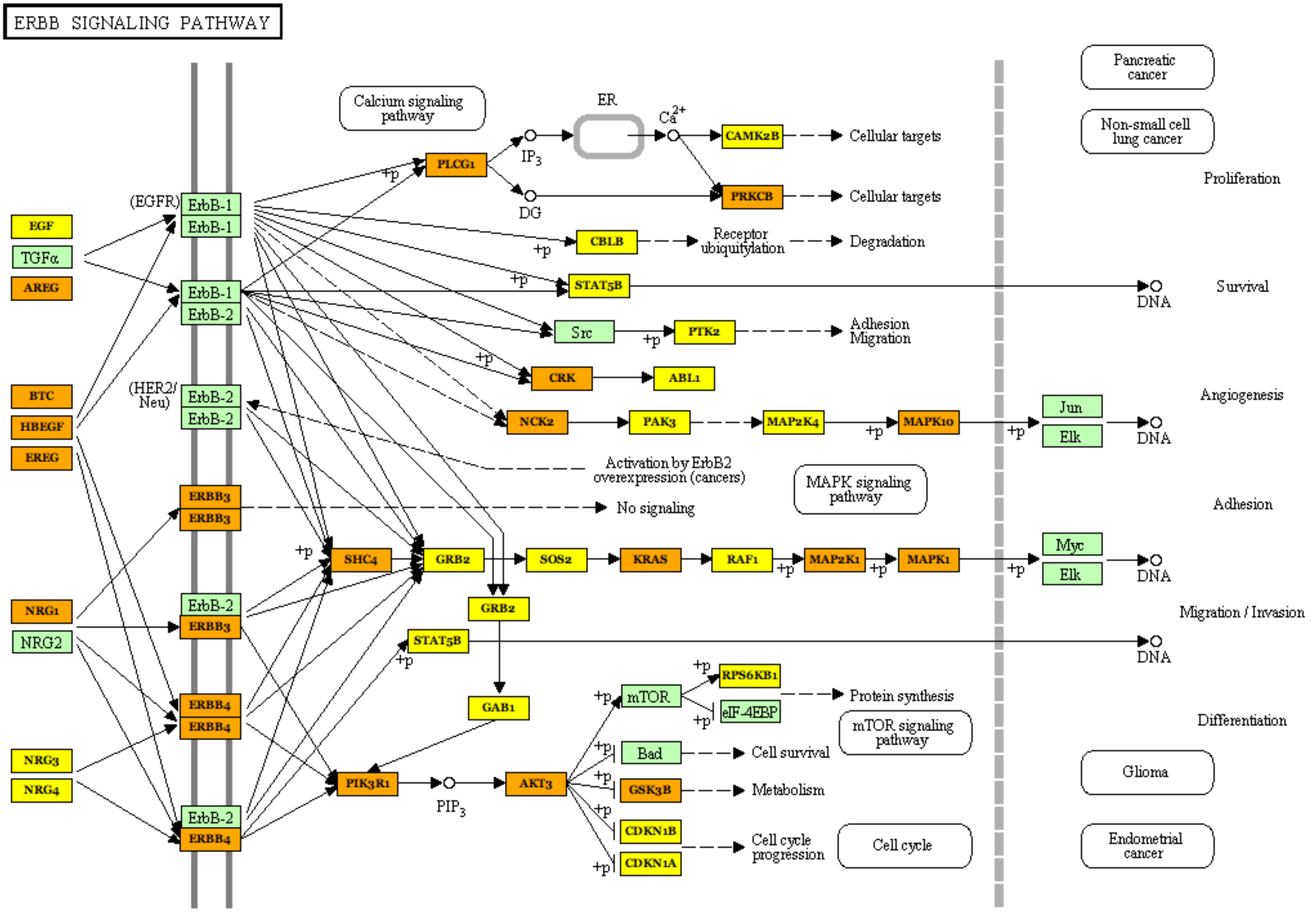

ERBB SIGNALING PATHWAY
Calcium signaling pathway
ER
Ca2+
IP3
DG
PLCG1
CAMK2B
PRKCB
Cellular targets
Cellular targets
CBLB
Receptor ubiquitylation
Degradation
STAT5B
DNA
Src
PTK2
Adhesion Migration
CRK
ABL1
NCK2
PAK3
MAP2K4
MAPK10
Jun
Elk
DNA
Activation by ErbB2 overexpression (cancers)
MAPK signaling pathway
No signaling
SHC4
GRB2
SOS2
KRAS
RAF1
MAP2K1
MAPK1
Myc
Elk
DNA
GRB2
STAT5B
DNA
GAB1
mTOR
RPS6KB1
eIF-4EBP
Protein synthesis
mTOR signaling pathway
Bad
Cell survival
PIK3R1
PIP3
AKT3
GSK3B
Metabolism
CDKN1B
CDKN1A
Cell cycle progression
Cell cycle
+p
(EGFR)
ErbB-1
ErbB-2
(HER2/ Neu)
ERBB3
ERBB4
EGF
TGFα
AREG
BTC
HBEGF
EREG
NRG1
NRG2
NRG3
NRG4
Pancreatic cancer
Non-small cell lung cancer
Glioma
Endometrial cancer
Proliferation
Survival
Angiogenesis
Adhesion
Migration / Invasion
Differentiation
